# Real-world outcomes from a series of patients with late onset Pompe disease who switched from alglucosidase alfa to avalglucosidase alfa

**DOI:** 10.3389/fgene.2024.1309146

**Published:** 2024-01-19

**Authors:** Chris Carter, Tracy Boggs, Laura E. Case, Priya Kishnani

**Affiliations:** ^1^ Division of Medical Genetics, Department of Pediatrics, Duke University Health System, Durham, NC, United States; ^2^ Department of Rehabilitation Services, Duke University Health System, Durham, NC, United States; ^3^ Doctor of Physical Therapy Division, Department of Orthopaedics, Duke University School of Medicine, Durham, NC, United States

**Keywords:** glycogen storage disease type 2, lysosomal storage disease (LSD), alglucosidase alfa, avalglucosidase alfa, late-onset Pompe disease (LOPD), enzyme replacement therapy

## Abstract

**Introduction:** Pompe disease is an inherited, progressive neuromuscular disorder caused by deficiency of lysosomal acid α-glucosidase and accumulation of glycogen in tissues, resulting in cellular dysfunction, muscle damage, and functional disabilities. Enzyme replacement therapy with alglucosidase alfa (Myozyme/Lumizyme) has led to better outcomes, but many patients have plateaued or declined despite treatment. The second-generation ERT avalglucosidase alfa (Nexviazyme) was designed to have enhanced cellular uptake via the conjugation of additional bis-mannose-6-phosphate residues. There have been trials comparing the efficacy of alglucosidase and avalglucosidase, but there remains a need for more real-world data on patients who switched from alglucosidase to avalglucosidase.

**Methods:** A chart review was conducted on *n* = 15 patients with late-onset Pompe disease followed at a single center who switched from alglucosidase to avalglucosidase and continued for at least 6 months.

**Results:** A total of *n* = 8/15 patients received alglucosidase for more than 3 years prior to switching, and *n* = 7/15 received it for more than 5 years prior to switching. There were statistically significant improvements in CK, Hex4, and AST with mean differences of −104.8 U/L, −3.0 mmol/molCr, and −14.7 U/L, respectively, post-switch. 6-Minute Walk Test; comfortable gait speed; Gait, Stairs, Gower, Chair; and Quick Motor Function Test scores improved or stabilized in most patients post-switch (*n* = 8/12, *n* = 11/12, *n* = 9/12, *n* =7/11, respectively). Of *n* = 7 patients with pulmonary function testing, *n* = 4/7 had improved upright FVC. Patient-reported outcomes revealed improvements in dyspnea (*n* = 4/4), physical function (*n* = 3/4), fatigue (*n* = 2/3), and lower back pain (*n* = 3/3). Avalglucosidase was well tolerated without infusion-associated reactions, and all *n* = 7 patients on home infusions continued receiving ERT at home. Anti-drug antibodies were seen in *n* = 9/10 of patients on alglucosidase and *n* = 8/13 of those on avalglucosidase, with titers below 12,800 in a majority of patients. We also present the first outcome data for a patient with LOPD who is non-ambulatory and a full-time wheelchair user; she demonstrated meaningful improvements in quality of life and motor function with the switch.

**Discussion:** In summary, improved outcomes were seen in most patients, with a subset whose decline persisted. This study presents evidence that switching from alglucosidase to avalglucosidase may be associated with improved outcomes in certain patients with LOPD.

## Introduction

Pompe disease, also known as glycogen storage disease type II (OMIM #232300), is an inherited, progressive neuromuscular disorder caused by deficiency of lysosomal acid ɑ-glucosidase (GAA) and accumulation of glycogen in various tissues, resulting in cellular dysfunction, progressive muscle damage, and functional disabilities.

Broadly, there are two categories based on age of onset and symptomatology: classic infantile-onset Pompe disease (IOPD) and late-onset Pompe disease (LOPD). IOPD is the most severe and rapidly progressive form of Pompe disease. Patients present with cardiomyopathy in the first year of life, and without treatment, fatal cardiorespiratory failure ensues with death in the first 1–2 years of life. Over the last few decades, prognoses have improved dramatically with the advent of newborn screening for Pompe disease and prompt initiation of treatment, preferably before the onset of severe signs and symptoms of the disease (Kishnani et al., 2021; Huggins et al., 2022). In contrast, LOPD includes cases of Pompe disease in which the age of onset is after 12 months, as well as those in which the age of onset is before 12 months who do not develop cardiomyopathy in the first year of life. Patients with LOPD experience progressive weakness in skeletal muscles including the diaphragm and other respiratory muscles, and consequently, respiratory failure remains the principal cause of death in LOPD (Toscano et al., 2019; Dimachkie et al., 2022).

LOPD may manifest in a wide spectrum of clinical presentations with varying ages of onset and rates of progression. The clinical diversity in LOPD can largely be explained by variable amounts of residual enzyme activity, considerable genotypic variability, and modifying factors that may have a sizeable effect on phenotype even among patients with the same variant (Toscano et al., 2019; Diaz-Manera et al., 2021).

Enzyme replacement therapy (ERT) with alglucosidase alfa was approved in 2006 as the first treatment for Pompe disease. Administering exogenous recombinant human GAA provides patients with an alternate means of cleaving glycogen, thus reducing accumulation and slowing disease progression. In IOPD and LOPD populations, ERT has remarkably improved both survival and quality of life (Schoser et al., 2017). For many years after its approval, alglucosidase alfa remained the standard of care for patients with Pompe disease. However, many patients on alglucosidase alfa infusions plateau or decline while on treatment, an indication that there remained an unmet need in the management of Pompe disease. This disease progression despite treatment has been partly attributed to suboptimal uptake of ERT into skeletal muscle (Diaz-Manera et al., 2021; Dimachkie et al., 2022).

A second-generation ERT, avalglucosidase alfa, was approved by the FDA in August 2021 for the treatment of patients 1 year of age and older with LOPD as well as by the European Commission in June 2022 for the treatment of both LOPD and IOPD. Avalglucosidase alfa has ∼15-fold more bis-mannose-6-phosphate (M6P) residues per enzyme molecule than alglucosidase alfa. This enhances uptake and trafficking to the lysosome via the cation independent M6P receptor, resulting in increased delivery, improved glycogen clearance, and better outcomes compared to alglucosidase alfa. In preclinical models, avalglucosidase alfa exhibited as much as five-times greater glycogen clearance compared to an equivalent dose of alglucosidase alfa (Zhu et al., 2009; Kishnani et al., 2021; Sanofi Genzyme, 2021).

According to the package insert, an avalglucosidase dosage of 20 mg/kg q2w is the standard recommendation for patients 30 kg or more, while 40 mg/kg q2w is recommended for those less than 30 kg (Sanofi Genzyme, 8/2021). This higher dose has also previously been shown in clinical trials (Mini COMET) to be safe and beneficial for patients with IOPD who showed clinical decline or a suboptimal response to alglucosidase alfa. Given the safety and efficacy of higher dosages in the IOPD population, some patients with LOPD who continued to decline despite 20 mg/kg q2w of avalglucosidase or were previously on 40 mg/kg/week of alglucosidase alfa were treated with 40 mg/kg q2w in this study. Further research into the safety and efficacy of increasing avalglucosidase dosage beyond standard recommendations is still needed.

Patients may develop antidrug antibodies (ADAs) against their ERT medications. These have long been associated with worse outcomes in IOPD, including declines in muscle strength, pulmonary function, and overall survival. There have also been reports of clinical decline in LOPD (de Vries et al., 2010; Desai et al., 2019; Kim et al., 2023). In general, persistent titers greater than or equal to 12,800 are viewed as clinically significant. Titers are monitored regularly, and patients with high and persistent titers often require immunomodulation (Sanofi Genzyme, 2010; Desai et al., 2019; Sanofi Genzyme, 2021; Kim et al., 2023).

In the real-world setting since the approval of avalglucosidase alfa, patients often switch from alglucosidase alfa to avalglucosidase alfa with the goal of improving clinical outcomes or preventing further decline. The difference in outcomes between these agents has been the subject of several studies with strict inclusion criteria and an emphasis on treatment-naïve patients (see Table 1), and there remains a paucity of evidence on the real-world results of switching from first-generation to second-generation ERT (Pena et al., 2019; Kishnani et al., 2021; Kishnani et al., 2023). Moreover, the switch data has been mostly limited to patients switching after relatively short durations of alglucosidase alfa; switch data from long-term ERT-experienced patients is scarce (Diaz-Manera et al., 2021; Dimachkie et al., 2022). Specifically, the COMET trial included *n* = 49 patients who switched after 49 weeks on alglucosidase alfa, and Dimachkie et al., 2022 included *n* = 10 patients who switched after 0.9–7 years and met criteria for inclusion. This study seeks to evaluate the clinical outcomes of patients who switched from alglucosidase alfa at various doses and variable durations to avalglucosidase alfa across a variety of outcome measures.

**TABLE 1:** Avalglucosidase in the literature. Notable studies comparing the efficacy of avalglucosidase alfa and alglucosidase alfa.

Study	Population, dosages	Results
Long-Term Safety and Efficacy of Avalglucosidase Alfa in Patients with Late-Onset Pompe Disease. Dimachkie, Mazen M., et al. (2022)	*n* = 7 treatment naïve, *n* = 10 switch patients (who received alglucosidase alfa for 0.9–7 years), all adults with LOPD. Doses of 5, 10, or 20 mg/kg q2w for 32–45 months and then 20 mg/kg q2w for all	Upright FVC and 6MWT distance remained stable in most participants, and improvements in 6MWT distance were observed in most participants <45 years at enrollment in both the naïve and switch groups
Safety and efficacy of avalglucosidase alfa versus alglucosidase alfa in patients with late-onset Pompe disease (COMET): a phase 3, randomised, multicentre trial. Diaz-Manera et al. (2021), Kishnani et al. (2023)	Patients ≥3 years of age with LOPD. *n* = 51 patients on avalglucosidase alfa, *n* = 49 on alglucosidase alfa in primary period. All on avalglucosidase alfa in extension. Doses of 20 mg/kg q2w	Outcomes at the primary endpoint of 49 weeks showed an increased mean improvement in percent predicted upright FVC on avalglucosidase alfa compared to alglucosidase alfa. This was sufficient to meet noninferiority criteria, but it narrowly missed the threshold for superiority (*p* = 0.063). Other findings include a greater increase in 6MWT distance on avalglucosidase alfa versus alglucosidase alfa. In the 49-week extension period, the results were consistent with the original findings demonstrating maintenance of positive clinical outcomes
Mini-Comet: Individual-Level Treatment Responses in Infantile-Onset Pompe Disease Participants Receiving Avalglucosidase Alfa or Alglucosidase Alfa Who Previously Received Alglucosidase Alfa. Kishnani, Priya, et al. (2021)	*n* = 22 patients with IOPD. Doses of 20 and 40 mg/kg q2w	Exploratory efficacy outcomes (6MWT, GMFM-88, QMFT, Pompe-PEDI, ptosis, and LVM *z*-score) improved or stabilized with avalglucosidase alfa at 40 mg/kg q2w, whereas these parameters stabilized or declined with 20 mg/kg q2w or on alglucosidase alfa

## Materials and methods

### Study design and population

This study was approved by the Duke Institutional Review Board under IRB number Pro00010830.

This study is a retrospective, longitudinal case series of patients seen at the Duke University metabolic clinic with a diagnosis of LOPD confirmed by molecular testing. Fifteen patients were identified who switched treatment from alglucosidase alfa to avalglucosidase alfa and continued for at least 6 months after the transition with varying amounts of post-switch clinical data. Patients, including patient 10, were excluded if they had no available follow-up data from at least 6 months after switching.

### Data collection

Data collection began from the first date in which the patient was seen in the Duke Health System and included all subsequent encounters prior to August 2023. Physical therapy (PT) data was collected through 11/13/2023. Information collected included demographics, medical history, and disease course, incorporating laboratory markers, antidrug antibodies, PT metrics, pulmonary function testing, and patient-reported outcomes. All data extracted from the medical record was compiled into a spreadsheet for analysis.

Patient-reported outcomes (PROs) were collected for Dyspnea (PROMIS Short Form v1.0–Dyspnea Severity–10a 01 August 2016), Physical Function (PROMIS Short Form v2.0–Physical Function 20a 29 November 2016), Fatigue (PROMIS Short Form v1.0–Fatigue–8a 16 March 2020), and Oswestry Low Back Pain Disability (Fairbank et al., 1980).

### Physical therapy outcome measures

All of the following outcome measures were performed by physical therapists experienced in neuromuscular disorders and the data obtained via chart review was evaluated by an experienced PT. The Six-Minute Walk Test (6MWT) was conducted to measure walking endurance per American Thoracic Society guidelines (ATS Committee on Proficiency Standards for Clinical Pulmonary Function Laboratories, 2002). Percent of predicted distance was calculated in accordance with norms established for age, gender, and height (Enright and Sherrill, 1998; Li et al., 2007). The Gait, Stairs, Gower, Chair (GSGC) test was used to measure motor function as previously described (Angelini et al., 2012; Khan et al., 2020a); GSGC scores range from 4 to 27, with a score of 4 indicating normal function. Patients were categorized into three degrees of impairment based on their GSGC score prior to the switch: mild (4–11), moderate (12–19), or severe (20–27). The Gross Motor Function Measure (GMFM-88) was used to test motor skills, with scores ranging from 0% to 100%, with 100% indicating normal function, which should be achieved by 5 years of age in those with typical function (Russell et al., 1989). The Quick Motor Function Test (QMFT), developed from the GMFM and validated in Pompe disease, has scores ranging from 0–64 with a score of 64 indicating normal function (van Capelle et al., 2012). Two subtests on the Bruininks-Oseretsky Test of Motor Proficiency Second Edition (BOT-2) were utilized: running speed and agility (subtest 6) and strength (subtest 8) (Deitz et al., 2007). For the patient who was non-ambulatory, the 9-Hole Peg Test (9-HPT) was used to measure hand dexterity (Wang et al., 2015), and lateral (key) pinch was used to assess strength as previously described (Paschall et al., 2021).

### Analysis

Descriptive analysis was used to summarize the baseline patient characteristics, treatments, and endpoints: Hex4, AST, CK, antidrug antibody titers, percent predicted forced vital capacity, PT outcome measures, and patient-reported outcomes. Statistical analysis and figure design were performed in GraphPad Prism 9. Paired t-tests were used in the analysis of laboratory markers between timepoints. For PT data, Intellectus Statistics (Intellectus Statistics 2019, Clearwater, Florida) was used to perform paired t-tests for examination of trends and to analyze differences between the pre- and post-switch assessments.

## Results

### Description of cohort and ERT history

Fifteen patients with LOPD were included in the study. *GAA* variants differed among the cohort, but the c.-32-13T>G variant predominated and was present in 12/15 patients in heterozygosity. The average age of onset in this cohort (Mean ± SD) was 27.75 ± 17.97, ranging from <1 year to 51 years of age. Muscle weakness was the most common presenting symptom. Fatigue, dyspnea, and myalgia were also frequently reported. *n* = 14/15 patients were ambulatory; patient 13 used both a power wheelchair and ventilator full-time. The median interval from symptom onset to diagnosis was 5.1 years (range 2.4 months–57 years). A full list of patient genotypes and other characteristics is included in Table 2.

**TABLE 2:** Summary of cohort characteristics. Note that patient 10 was excluded from the study due to a lack of data.

Patient/Sex	*GAA* genotype	Onset age (y)	Diagnosis age (y)	Primary symptoms
1/F	c.-32-13T>G	48	56	Muscle weakness, atrophy, myalgia, gait disturbance, poor endurance
	c.1796C>A			
2/F	c.-32-13T>G	32	34	Muscle weakness, myalgia, dyspnea, gait disturbance, fatigue, dysphagia
	c.1445C>G			
3/F	c.2481 + 110_2646 + 39del	40	43	Muscle weakness, atrophy, fatigue, dyspnea, lower back pain
	c.2294A>G			
4/M	c.-32-13T>G	20	77	Muscle weakness, gait disturbance, loss of agility, fatigue
	c.1655T>C			
5/F	c.-32-13T>G	30	48	Muscle weakness, loss of balance, hypophonia, fatigue, dysphagia
	c.1548G>A			
6/F	c.-32-13T>G	51	71	Muscle weakness, dyspnea, gait disturbance, loss of balance, fatigue
	c.1943G>A			
7/F	c.525delT	47	67	Muscle weakness, dyspnea, fatigue, dysphagia
	c.336–13T>G			
8/M	c.-32-13T>G	27	44	Muscle weakness, dyspnea, gait disturbance, scoliosis, fatigue
	c.1827delC			
9/M	c.-32-13T>G	43	43	Muscle weakness, lower back pain, fatigue
	c.1143delC			
11/F	c.-32-13T>G	16	16	Muscle weakness, myalgia
	c.2236T>C			
12/F	c.-32-13T>G	49	49	Muscle weakness, dyspnea, gait disturbance, scoliosis, dysphagia
	c.1572C>A			
13/F	c.1655T>C	<1	1.75	Delayed motor milestones, poor head control, low tone, wheelchair and ventilator dependence
	c.896T>G			
14/M	c.-32-13T>G	15	46	Muscle weakness, myalgia, lower back pain, fatigue
	c.1942G>A			
15/M	c.-32-13T>G	2	2.5	Muscle weakness, myalgia, gait disturbance, delayed motor skills, fatigue
	c.1051delG			
16/M	c.-32-13T>G	2	2	Muscle weakness, gait disturbance, delayed motor skills, scoliosis
	c.2501_2502delCA			

Median interval from diagnosis to ERT initiation was 2.5 months (range 0.3 months–8 years). Patients received alglucosidase alfa for periods ranging from 2.3 months to 19.1 years with a median duration of 3.8 years. A total of *n* = 8/15 patients received alglucosidase alfa for more than 3 years, and *n* = 7/15 received it for more than 5 years. Patients 2, 4, 8, and 13 underwent dosage increases during their time on alglucosidase alfa. At the time of switching to avalglucosidase alfa, patients were on alglucosidase alfa dosages ranging from 20 mg/kg q2w to 40 mg/kg q1w.

Median age of switch was 52 years. *n* = 11 patients started avalglucosidase alfa at 20 mg/kg q2w, *n* = 3 (patients 4, 8, and 13) started at 40 mg/kg q2w, and *n* = 1 (patient 5) started at 5 mg/kg q2w as part of the COMET trial before increasing to 20 mg/kg q2w. Patient 7 had an avalglucosidase alfa dose increase during her treatment from 20 to 40 mg/kg q2w. At the time of database lock, all patients continued to receive avalglucosidase alfa and have received it for durations ranging from 10 months to 9.9 years (median 18.9 months).

**FIGURE 1:**
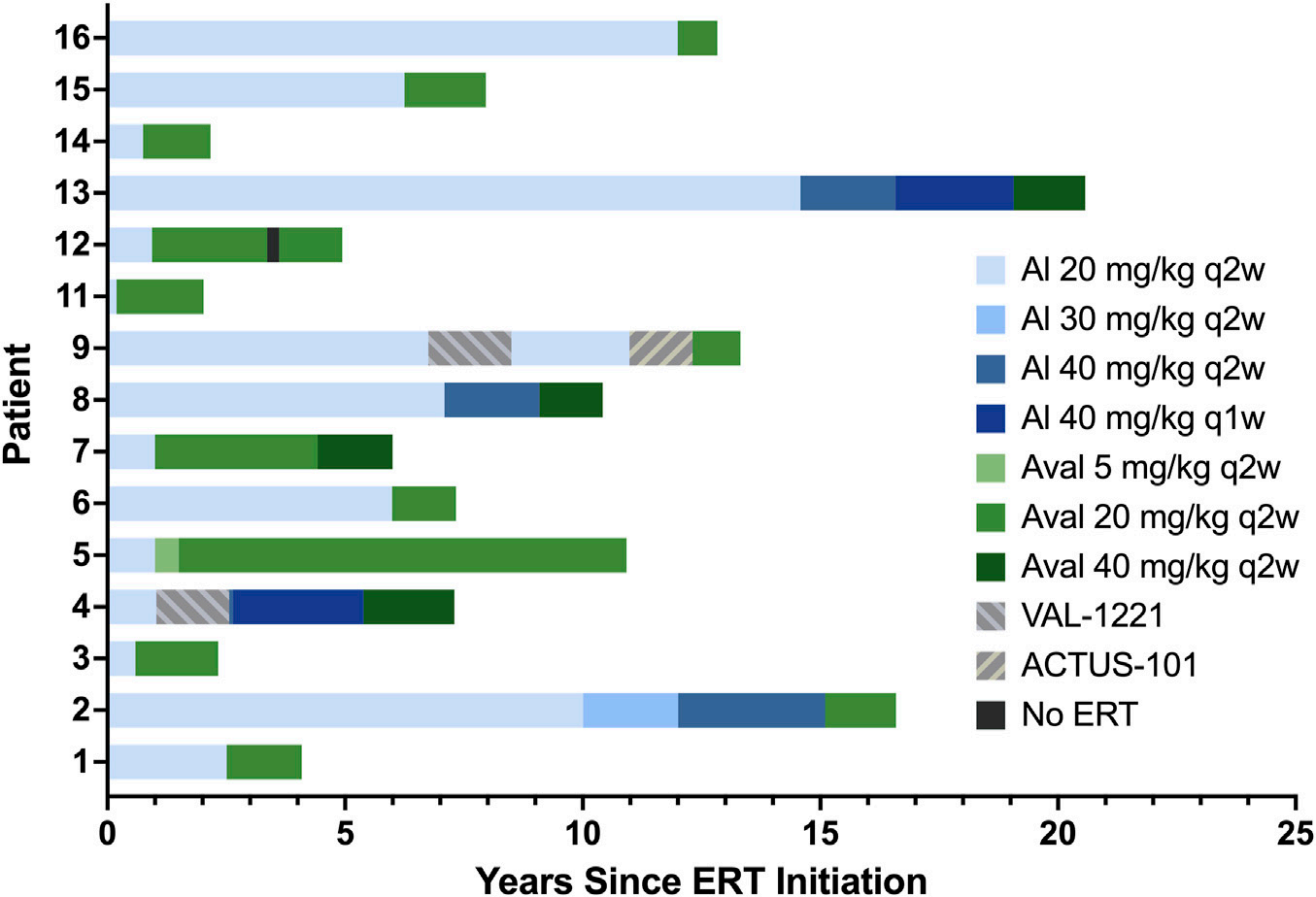
ERT history. Alglucosidase regimens are indicated by shades of blue, avalglucosidase regimens are indicated by shades of green, and other regimens are indicated by patterned gray bars.

Patients 4 and 9 stopped alglucosidase therapy for 17 and 21 months, respectively, while participating in the VAL-1221 clinical trial. Both of them withdrew due to a lack of clinical benefit and resumed alglucosidase therapy for 34 and 30 months, respectively, before switching to avalglucosidase. Patient 9 also stopped receiving alglucosidase to participate in a gene therapy trial for 16 months; after withdrawing from that study due to lack of clinical benefit, he switched to avalglucosidase therapy. Finally, patient 12 stopped avalglucosidase therapy for 3 months mid-treatment due to insurance issues. Full treatment history is detailed in Table 3 and depicted in Figure 1.

**TABLE 3:** Treatment history. All dosages are 20 mg/kg q2w unless otherwise specified.

Patient/Sex	Age at ERT initiation (y)	Time on alglucosidase (mo)	Age at ERT switch (y)	Time on avalglucosidase (mo)	Notes
1/F	57	30	59	19	
2/F	42	121 (20 mg/kg q2w)	58	18	
		23 (30 mg/kg q2w)			
		37 (40 mg/kg q2w)			
3/F	43	7	44	21	
4/M	77	12 (20 mg/kg q2w)	82	23 (40 mg/kg q2w)	17 months VAL-1221 after 12 months on al
		1 (40 mg/kg q2w)			
		33 (40 mg/kg q1w)			
5/F	48	12	49	6 (5 mg/kg q2w)	
				113 (20 mg/kg q2w)	
6/F	71	72	77	16	
7/F	67	12	68	41 (20 mg/kg q2w)	
				19 (40 mg/kg q2w)	
8/M	44	85 (20 mg/kg q2w)	53	16 (40 mg/kg q2w)	
		24 (40 mg/kg q2w)			
9/M	45	81 (20 mg/kg q2w)	58	12	21 months VAL-1221 after 81 months on al
		30 (20 mg/kg q2w)			16 months ACTUS-101 before switch
11/F	16	2.3	16	22	
12/F	51	11	52	29 (20 mg/kg q2w)	3 months stopped ERT after 29 months on aval
				16 (20 mg/kg q2w)	
13/F	7	175 (20 mg/kg q2w)	25	18 (40 mg/kg q2w)	
		24 (40 mg/kg q2w)			
		30 (40 mg/kg q1w)			
14/M	46	9	47	17	
15/M	2.5	75	9	21	
16/M	2	146	14	10	

### Laboratory markers

**FIGURE 2:**
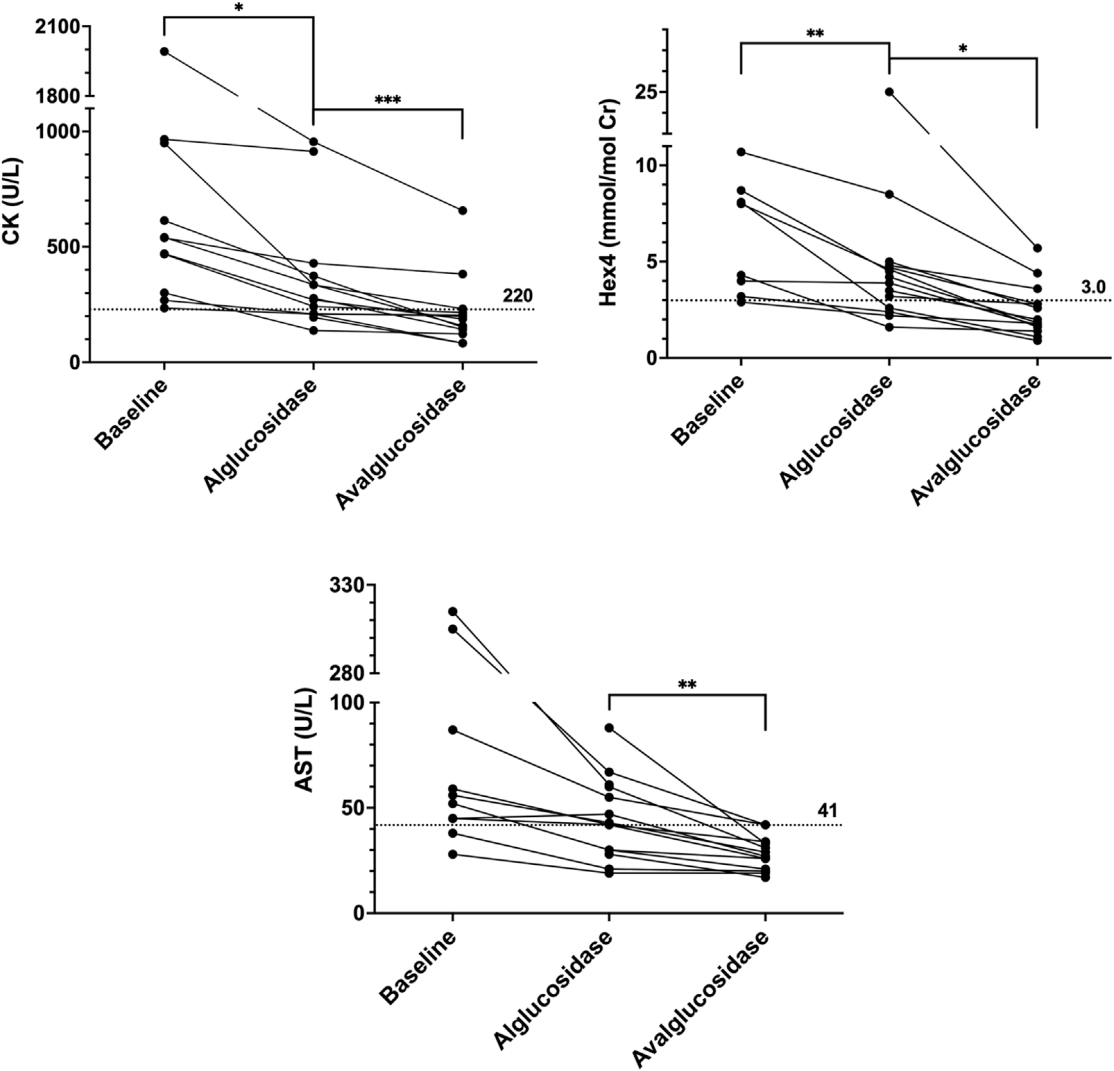
CK, Hex4, and AST trends. Baseline values are the last known values prior to initiating ERT. Alglucosidase values are the last known values prior to the switch. Avalglucosidase values are the most recent values available in patient’s chart. Dotted horizontal lines are upper limits of normal.

When available, urinary Hex4 (Glc4) and serum CK, AST, and ALT values were obtained via chart review at three time points: at baseline prior to ERT initiation, while on alglucosidase prior to ERT switch, and while on avalglucosidase at the most recent assessment. These values were compared to all other timepoints to ensure that they were representative of overall trends. Trends are depicted in Figure 2 and full data is available in Supplementary Tables S1, S2; Supplementary Figure S1.

From baseline to alglucosidase alfa, all patients showed a reduction or stabilization across all biomarkers. Average differences (Mean ± SD) in CK, AST, ALT, and Hex4 were −266.5 ± 301.2 U/L, −60.3 ± 98.40 U/L, −51.9 ± 74.78, and −2.449 ± 1.876 mmol/mol Cr, respectively. Changes in CK and Hex4 were statistically significant (*p* = 0.0149 and *p* = 0.0077, respectively). Expressed as a percent drop from baseline to ERT with alglucosidase alfa, the changes were −44% (CK), −56% (AST), −49% (ALT), and −10% (Hex4).

A further reduction or stabilization in CK, AST, ALT, and Hex4 was seen from alglucosidase alfa to avalglucosidase alfa in all patients. Average differences from alglucosidase to avalglucosidase (Mean ± SD) in CK, AST, ALT, and Hex4 were −104.8 ± 89.01 U/L, −14.7 ± 14.58 U/L, −22.1 ± 26.55 U/L, and −2.998 ± 4.811 mmol/mol Cr, respectively. Changes in all four biomarkers were statistically significant (*p* = 0.0011 for CK, *p* = 0.0023 for AST, *p* = 0.0083 for ALT, and *p* = 0.0364 for Hex4). Expressed as a percent drop from pre-to post-switch, the changes were −40% (CK), −39% (AST), −43% (ALT), and −56% (Hex4).

Across all biomarkers, there was also a considerable increase in the number of values that fell below the upper limit of normal. This was the case when comparing values prior to starting any ERT to those while on alglucosidase, as well as comparing values on alglucosidase to those on avalglucosidase. Across the three timepoints, the number of CK values less than 220 U/L increased from 0 to 5 to 11. The same trend was seen with AST, from 1 to 6 to 12 values less than 41 U/L, and ALT, from 2 to 6 to 13 values less than 40 U/L. Finally, this was also the case with Hex4, which went from 1 to 4 to 11 values that fell below 3.0 mmol/mol Cr.

### Physical therapy measures

Thirteen of the 15 patients in this study had PT data available from before and after the switch to avalglucosidase alfa: 12 were ambulatory (patients 1–7, 9, 12, 14–16) and one was non-ambulatory (patient 13, discussed separately below). Differences between the last pre-switch assessment and the most recent assessment were analyzed for each performance metric. An overview of PT outcomes with patients categorized into groups of severity is included in Table 4.

**TABLE 4:** PT outcomes at most recent visit. Patients were categorized based on GSGC score prior to the switch as having mild (GSGC 4–11, green), moderate (GSGC 12–19, yellow), or severe (GSGC 20–27, red) impairment. Notably, patients who declined on avalglucosidase were more likely to be those with severe impairments, while those with moderate and mild impairments were generally more likely to stabilize or improve. An MDC of 5% was used to qualify as a change in 6MWT distance, and an MCID of 18.9% change was used for gait speed. Patients with any increase in QMFT, GMFM, and BOT2 were placed in the improved category, and any decrease in score was considered a decline. For GSGC, any increase in score was considered a decline, and any decrease in score was considered improved.

	6MWT	GSGC	Comfortable gait speed	QMFT	GMFM	BOT2
Improved	3F, 9M, 16M	15M	15M, 16M	1F, 15M, 16M	15M	16M
	5F	12F	2F, 5F	2F		
	4M		4M, 6F	12F		
Stable	1F, 14M	1F, 3F, 9M, 16M	1F, 3F, 9M, 14M	3F, 9M	9M, 16M	–
	2F	2F	12F			
		4M, 6F				
Declined	15M	14M	7F	14M	–	–
	6F, 7F, 12F	5F		6F, 4M, 7F		
		7F				

### 6MWT

At the most recent post-switch assessment, there were no trends or statistically significant differences in the mean distance walked for the 6MWT or in the percent predicted, whether analyzed as a group or by level of severity.

However, looking at individual 6MWT performances at the most recent visit, 8/12 (67%) did show an increase in distance walked (mean increase 39.4, range 2.4–80.2 m) while 4/12 (33%) showed a decrease in distance walked (mean decrease −72.7, range −84.3 to −50.0 m). Using a minimal detectable change (MDC) cutoff of 5% change in distance walked for the 6MWT (Claeys et al., 2022), 5/12 patients (41.7%) showed an increase greater than the MDC, 3/12 (25%) showed improvements that did not meet the MDC, and 4/12 (33.3%) had a decrease greater than the MDC.

To look at clinically significant changes on the 6MWT at the individual level, a minimal clinically important difference (MCID) of 30 m was used, as suggested for adults with neuromuscular disorders (but not Pompe-specific) (van der Ploeg et al., 2010; Bohannon and Crouch, 2017). At the most recent visit, 5/12 participants (41.7%) showed a clinically important increase in distance walked (mean increase 60.5, range 33.6–80.2 m), while 3/12 (25%) improved but did not show a clinically important difference (mean increase 18.4, range 2.4–21.6 m), and 4/12 (33.3%) showed a clinically important decrease in distance walked (mean decrease −72.7, range −84.3 to −50.0 m).

Full 6MWT trends are included in Supplementary Figure S2.

### GSGC

For the total GSGC (qualitative) score, 2/12 (16.7%) showed improvement (both by 2 points), 7/12 (58.3%) remained stable (no change in score), and 3/12 (25%) declined (by an average of 1.25, range 1–3) at their most recent visit. Full GSGC trends are included in Supplementary Figure S3. While most patients showed stability in overall GSGC score at each of the post-switch time points, there were notable trends of improvement in the time it took to walk 10 m (Gait). At the most recent visit, there were no statistically significant changes (alpha of 0.05) in the Stairs, Gowers, or Chair quantitative (timed) scores.

There is no published MDC or MCID for gait speed in Pompe, but using an MDC of 0.15 m/s (Middleton et al., 2015) as a cutoff, comfortable (self-selected) gait speed improved in 5/12 (41.7%) patients (mean improvement 0.33, range 0.24–0.47 m/s), remained stable in 7/12 (58.3%) (mean change 0.04, range −0.12–0.14 m/s), and declined in none at their most recent visit. When applying the gait speed cutoffs for Duchenne muscular dystrophy, given its similar gait presentation to Pompe, 6/12 patients met the lower MCID of 18.9% of baseline indicating clinically significant improvement, and 4/12 met the higher bar of 31.1% (McDonald et al., 2013). Two patients (P4, P7) had changes that were significant using both MCID cutoffs, but their baseline walking speeds were low enough that these cutoffs were lower than the MDC of 0.15 m/s. Patient 4 had significant improvement (+0.10 m/s) and patient 7 had a significant decline (−0.12 m/s).

For the whole group, both the mean comfortable gait speed and mean fast gait speed were faster at the most recent visit post-switch, showing statistical significance at an alpha level of 0.05 (comfortable gait speed pre-switch mean 0.90, range 0.32–1.36 m/s, most recent mean 1.05, range 0.20–1.41 m/s; fast gait speed pre-switch mean 2.13 m/s, range 1.49–2.76, most recent mean 2.48 m/s, range 1.61–3.73). See Supplementary Figures S4, S5 and Supplementary Table S7 for full comfortable and fast gait speed data.

### QMFT

Eleven of the twelve patients had QMFT data; of these, 5/11 (45%) had improved scores, 2/11 (18%) remained stable with no change in score, and 4/11 (36%) had scores that declined at their most recent visit. The average increase in those who improved was 5 points (range 1–11), while the average decrease in decliners was −2.75 points (range −5 to −1). No MDC has been established for the QMFT. Full QMFT data including individual item response is detailed in Supplementary Table S5.

### GMFM and BOT-2

Only three patients (P9, P15, P16) had GMFM data, and all were seen only once after the switch. The total GMFM score improved from 98.86% to 100% for one patient and remained unchanged for the other two. One patient (P16) had BOT-2 scores and his score improved on the running speed/agility scale score from 12 to 14, improved on the strength and agility standard score from 43 to 46 (24th to 35th percentile), and remained unchanged on the strength scale score at 11. For both of these subtests, he placed in the “average” descriptive category before and after the switch.

### Pulmonary function testing

**FIGURE 3:**
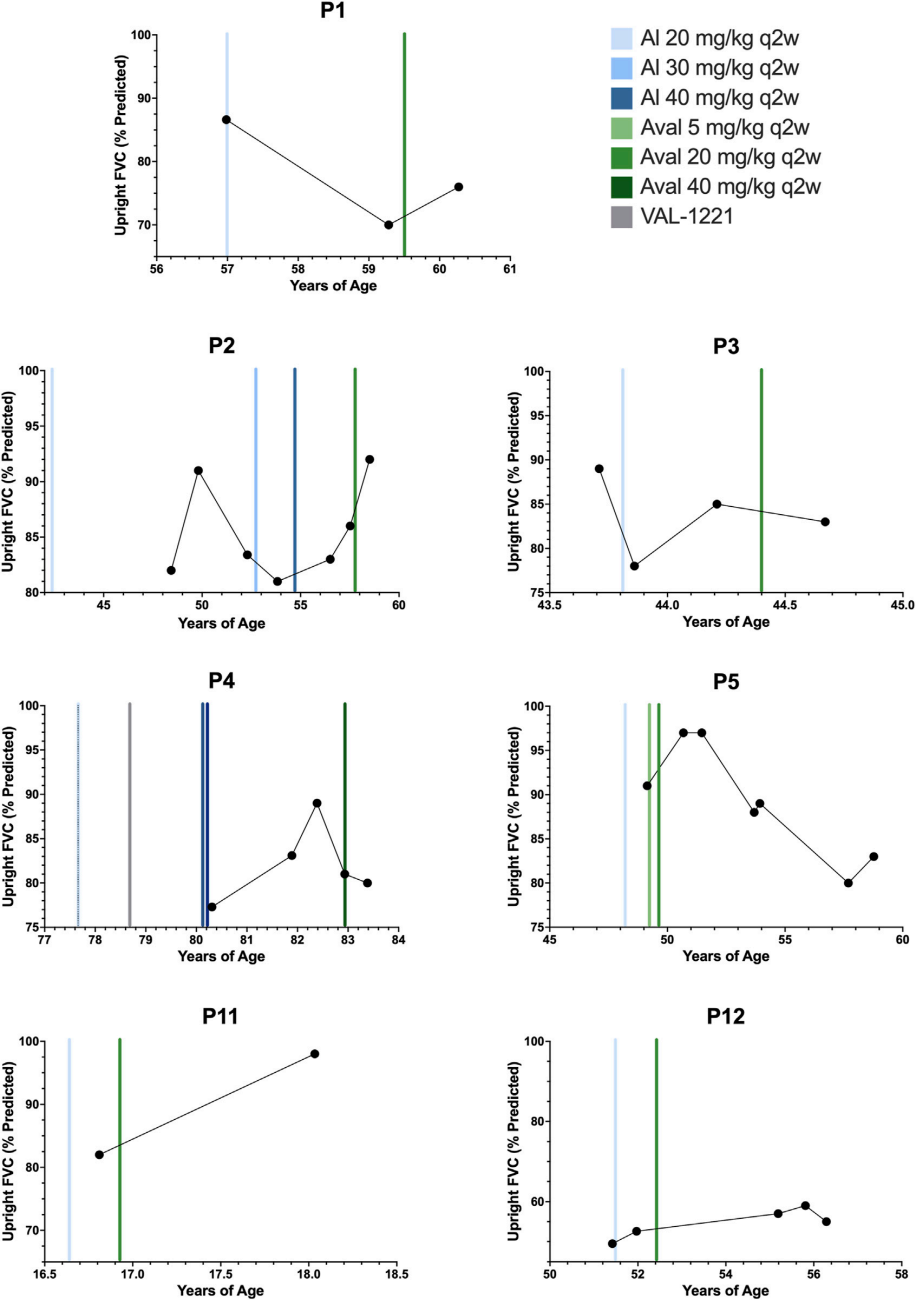
Upright forced vital capacity. Percent predicted upright forced vital capacity results for patients 1, 2, 3, 4, 5, 11, and 12. Color-coded vertical bars represent the starting points of ERT regimens.

Longitudinal pulmonary function test (PFT) data was available for *n* = 7 patients (P1-5, P11, P12) and is depicted in Figure 3. To our knowledge, no specific minimal clinically important change (MCIC) for PFTs in Pompe disease has been established (Lachmann and Schoser, 2013).

Of *n* = 7 patients, *n* = 4/7 (P1, P2, P11, P12) experienced improvement in percent predicted upright forced vital capacity (FVC) after switching to avalglucosidase alfa with an average improvement (Mean ± SD) of +7.6% ± 5.9%. The remaining *n* = 3/7 patients (P3, P4, P5) experienced decreases. The average decrease was −3.7% ± 3.8%. Overall, the mean change in percent predicted FVC was +2.7 ± 7.6%. The median interval between baseline PFT testing on alglucosidase and most recent evaluation on avalglucosidase was 12 months, ranging from 5.4 months to 9.6 years. Notably, one patient who declined (P5) did demonstrate improvements in FVC for over 2 years and peaked at 97% before declining through her most recent evaluation to 83% at 9.6 years post-switch.

### Patient-reported outcomes

**FIGURE 4:**
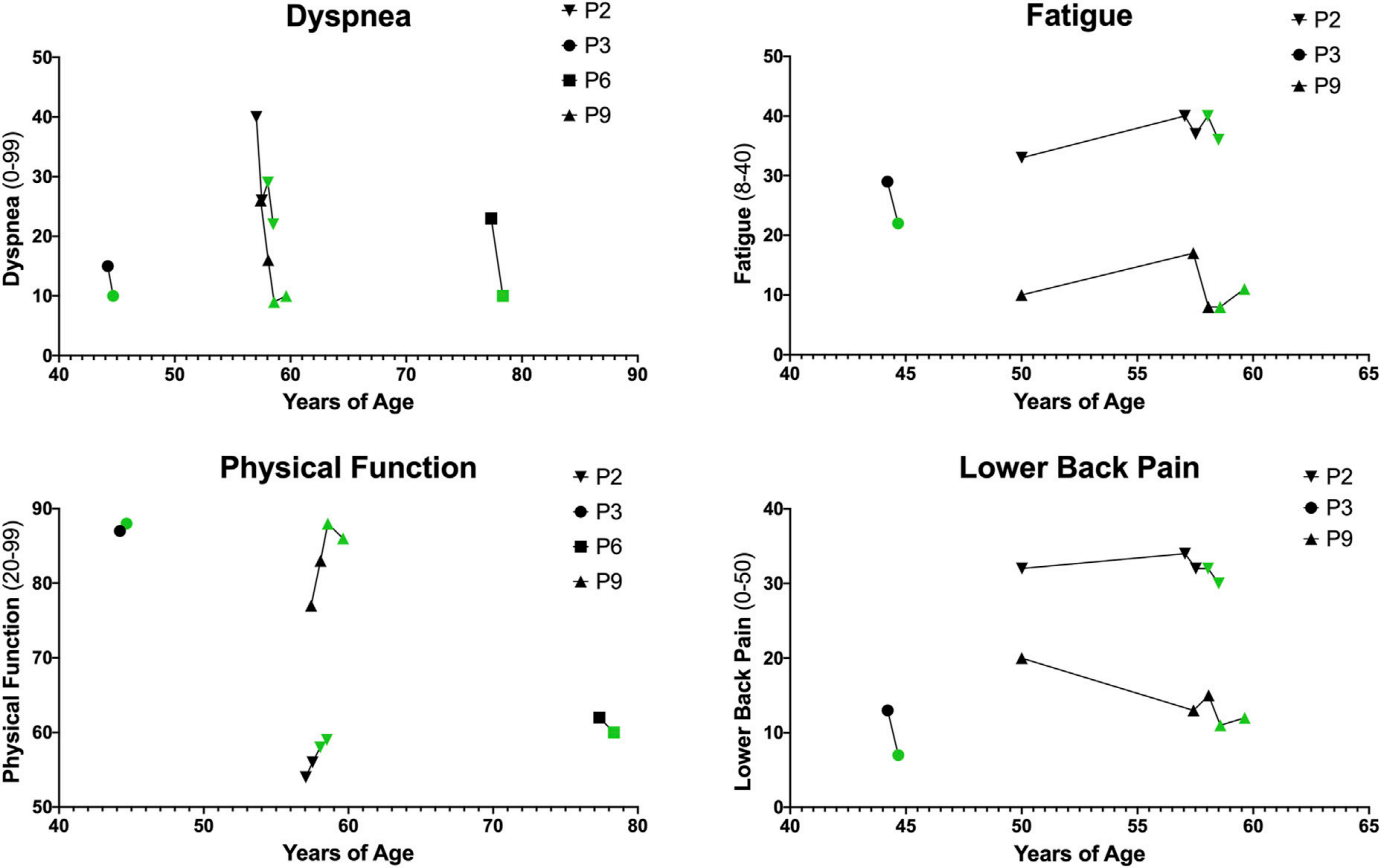
Patient-Reported Outcomes (PROs). PROs for dyspnea, physical function, fatigue, and lower back pain. Higher dyspnea scores (range 0–99) indicate increased severity of dyspnea with common daily activities. Higher physical function scores (range 20–99) indicate increased physical function and ability to carry out daily life activities. Higher fatigue scores (range 8–40) indicate increased fatigue on average over the past week. Higher lower back pain scores (range 0–50) indicate greater severity of lower back pain and impact on life.

Patient-reported outcome (PRO) data was available for *n* = 4 patients (P2, P3, P6, and P9). All PRO data for dyspnea, physical function, fatigue, and lower back pain is depicted in Figure 4.

Dyspnea is reported on a scale from 0 to 99 with higher scores indicating increased severity of dyspnea with common daily activities. Dyspnea improved in *n* = 4/4 patients with an average (Mean ± SD) difference of −7.00 ± 4.08. Physical function is reported on a scale from 20 to 99 with higher scores indicating increased physical function and ability to carry out daily life activities. Physical function improved in *n* = 3/4 patients with an average difference of 1.25 ± 2.36. Fatigue is reported on a scale from 8 to 40 with higher scores indicating increased fatigue on average over the past week. Fatigue improved in *n* = 2/3 patients and with an average difference of −1.67 ± 5.03. Lower back pain, reported via the Oswestry Disability Index, ranges from 0 to 50 with higher scores indicating greater severity of lower back pain and disability. In this cohort, *n* = 3/3 patients reported improved back pain with an average difference of −3.67 ± 2.08.

### Safety outcomes and antidrug antibodies

There were five reports of infusion reactions while receiving alglucosidase alfa. Patient 2 reported experiencing intermittent diarrhea with infusions. Patient 6 reported an event in which she experienced epigastric pain, tongue swelling, and uncontrollable salivation during an alglucosidase infusion. This resolved with diphenhydramine. Patient 13 reported repeated episodes in which she experienced a full-body rash, emotional difficulties, bloating, body pain, hand/body tremors, nausea, and fatigue. These were associated with the switch to more frequent (weekly) and longer (7–7.5 h) alglucosidase alfa infusions, and they resolved with the switch to avalglucosidase alfa. Finally, patients 7 and 12 reported mild reactions of chills and itching, respectively. There was one report of infusion reaction in this cohort after the change to avalglucosidase alfa; patient 11 reported two episodes of fever associated with infusions. All *n* = 7 patients on home infusions of alglucosidase alfa were able to continue home infusions for avalglucosidase alfa, and *n* = 3 additional patients switched to home infusions after switching ERT.

Most recent and peak ADA titers for all patients are listed in Supplementary Tables S3, S4, and full titer history for each patient is detailed in Supplementary Figure S1. Out of *n* = 10 patients with data available while on alglucosidase alfa, *n* = 9/10 had positive ADA titers. All positive titers fell below 12,800 except for patients 6 and 9, who both peaked at 12,800. Patient 9 only reached this level at one timepoint, while patient 6 reached this level three times over the course of 5 years. At the time of switching, P6 had a titer of 12,800 and P9 had a titer of 200.

Out of *n* = 13 patients with ADA data available while on avalglucosidase alfa, *n* = 8/13 had positive titers. Notably, patient 6 had negative titers while on avalglucosidase. All positive titers fell below 12,800 except for patients 11 and 14, who peaked at 102,400 and 25,600, respectively. Patient 11 has three ADA data points while on avalglucosidase alfa, and these are trending downward (102,400, 51,200, 25,600), most recently 20.34 months post-switch. Patient 14 had two ADA data points (12,800 and 25,600) since switching to avalglucosidase alfa, most recently 12.39 months post-switch.

Patient 11 was on alglucosidase for 2.3 months prior to switching, and she had no ADA titer data from this period. Since the switch, she has shown an increase in percent predicted FVC of 16%, though this is limited to two available time points. Her biomarkers showed improvements of 11% in CK and AST, both of which normalized, as well as an improvement of 63% in Hex4, which has remained within normal limits. She has had no infusion-associated reactions. Patient 14 was on alglucosidase for 9 months prior to switching and had titers of 6,400 at 3.71 months before switching. For patient 14, PT assessment showed stability in 6MWT and gait speed with a worsening of GSGC and QMFT by one point each. Biomarkers in this patient showed improvements of 31% in CK, 56% in Hex4, and 24% in AST. CK (657 U/L) and AST (42 U/L) remained above the upper limit of normal, while Hex4 normalized. He has also had no infusion-associated reactions. Overall, limited post-switch data in patients 11 and 14 present a mixed bag of outcomes, and the impact of these elevated titers is unclear at this time. Continued close follow-up is necessary to evaluate their effects.

### Patient 13: non-ambulatory case

Patient 13 represents the first reported case of a patient with LOPD who is non-ambulatory and switched from alglucosidase alfa to avalglucosidase alfa. She was diagnosed at age 21 months and retrospectively reported symptom onset within the first year of life without cardiomyopathy. Her initial symptoms were low tone, decreased head control, and delayed achievement of motor milestones. From a motor standpoint, she was able to independently drive her motorized wheelchair using a joystick at her most recent assessment, but she has been dependent for all functional mobility since 3 years of age and requires full-time care. She was initially managed with nasal intermittent positive pressure ventilation but progressed to tracheostomy tube and ventilator dependence by age 4. Her past medical history is also significant for T4-sacral posterior spinal fusion surgery, oral-maxillary reconstructive surgery, history of fractures secondary to severe osteoporosis, and severe recurrent kidney stones secondary to poor water intake. She received alglucosidase alfa infusions for 19 years, up to a maximum dose of 40 mg/kg q1w. Due to a combination of lack of response to alglucosidase, infusion-associated reactions (full-body rash, emotional difficulties, myalgias, nausea, fatigue), and prolonged infusion time (7–7.5 h), she switched to avalglucosidase alfa at 40 mg/kg q2w. From her perspective, she reported improvements on avalglucosidase that far outweigh the minimal, if any, improvements that she noted on alglucosidase alfa.

Her hand dexterity was measured using the 9-HPT by her local occupational therapist. She was unable to complete the standard 9-HPT prior to switching but was able to complete a modified version using easy grip pegs. She was making improvements prior to the switch and demonstrated continued improvements in her right hand after the switch, from 110 s prior to switch to 87 s on day 12 and 57 s on day 286. She also demonstrated continued improvements in her left hand, from 162 s prior to the switch to 110 s on day 12 and 59 s on day 286. Furthermore, by day 286, she had also improved enough to complete the standard 9-HPT with her right hand, improving from 98 s on day 286 to 58 s on day 518 (norm is 16.04 s ± 1.82) (Oxford Grice et al., 2003). She was able to complete the task with her left hand by day 356, but a time was not documented. She was only able to complete part of the test with her left hand on day 433, which was attributed to edema and pain noted in her arm, but by day 518, she was again able to complete it with her left hand in 116 s. Full 9-HPT details are available in Supplementary Table S6. She did not have baseline pinch data, but when tested, she demonstrated improved lateral pinch from day 290 to day 465 from 3.83 to 4.0 lbs on right and from 2.77 to 3.17 lbs on left, respectively (right hand norm 17.7 ± 2.1 lbs and left hand norm 16.6 ± 2.1 lbs) (Mathiowetz et al., 1985).

Subjectively, she and her mother reported improvements in hand function, head control, breathing, and endurance, all substantiated by her local OT and PT. Her reported fatigue with school activities and transfers decreased from 9/10 to 5/10. She reported an improved ability to raise a utensil to her mouth, move her cell phone from her lap to her armrest, use a keyboard, and flip her notebook. Remarkably, she also reported being able to chew longer without fatigue, suck through a straw, and blow out her birthday candles for both birthdays since switching. She also reported being able to breathe for 5 min off of her ventilator, which she has not been able to do for 12–13 years. Overall, she reported having more energy, less brain fog, and feeling happier.

## Discussion

For many years after its approval, alglucosidase alfa remained the standard of care for patients with Pompe disease. The treatment has been lifesaving, but many patients on alglucosidase alfa infusions continue to decline while on treatment and require increased dosages, an indication that there remained an unmet need in the management of Pompe disease (Khan et al., 2020b; Diaz-Manera et al., 2021; Dimachkie et al., 2022). Second-generation ERT with avalglucosidase alfa was designed to exhibit enhanced uptake in target cells, thereby improving clinical response. Over the last few years, several studies have provided evidence of improved outcomes with avalglucosidase, though these studies used strict inclusion criteria and focused primarily on treatment-naïve patients. The COMET trial included *n* = 49 patients who switched after just 49 weeks on alglucosidase alfa, and Dimachkie et al., 2022 included *n* = 10 patients who switched after 0.9–7 years (Diaz-Manera et al., 2021; Kishnani et al., 2021; Dimachkie et al., 2022). This study describes real-world experiences in patients with LOPD, including *n* = 8 on alglucosidase alfa for more than 3 years, *n* = 7 for more than 5 years. The data generated provides additional evidence that switching from alglucosidase alfa to avalglucosidase alfa may lead to better outcomes across a variety of measures.

Our cohort of fifteen patients includes a diversity of genotypes and presentations, incorporating patients across a wide age range and broad spectrum of severity. We report data across a variety of measures and discuss the first report of outcomes when switching from alglucosidase to avalglucosidase in a patient with LOPD who uses a power wheelchair and ventilator full-time. Dosages and durations of ERT regimens varied widely among the cohort. *n* = 8/15 patients received alglucosidase alfa for greater than 3 years before switching, and *n* = 7/15 received it for greater than 5 years. Notably, we present four patients (P4, P7, P8, P13) receiving avalglucosidase at doses of 40 mg/kg q2w. We also present one patient (P2) who switched from alglucosidase alfa at a dose of 40 mg/kg q2w to avalglucosidase alfa at 20 mg/kg q2w and demonstrated improvement. Patients 4, 9, and 12 also stood out from the others, as patients 4 and 9 have a history of enrollment in clinical trials, and patient 12 stopped ERT for 3 months due to insurance issues. Enrollment in these clinical trials did not appear to have any positive impact on the clinical course of these patients; both continued to decline and withdrew from the trials before 2 years of enrollment. Despite all of these differences in ERT regimens, these patients demonstrated improvement across most outcomes (see overview of outcomes in Table 5).

**TABLE 5:** Overall outcome heat map. Changes in all outcomes organized by disease severity and color-coded (improvement in green, stability in blue, decline in red). Number of months on alglucosidase and avalglucosidase (at any dose) included. Laboratory values that remain elevated beyond the reference range after the switch to avalglucosidase have been bolded.

		P1	P3	P9	P11	P14	P15	P16	P2	P5	P4	P6	P7	P8	P12	P13
Severity		Mild	Mild	Mild	Mild	Mild	Mild	Mild	Mod	Mod	Sev	Sev	Sev	Sev	Sev	Sev
Al. Months		30	7	111	2.3	9	75	146	181	12	46	72	12	109	11	229
Aval. Months		15	17	8	18	13	17	6	14	115	19	12	56	12	41	14
Labs	CK (%)	−53	−**11**	−31	−11	−**31**	−**31**	–	−49	−59	−11	−57	−5	−2	−60	–
	Hex4 (%)	−56	−58	−13	−63	−56	−48	−13	−40	−65	−18	−43	−**25**	–	−**48**	−**77**
	AST (%)	−33	−19	−13	−11	−**24**	−**37**	–	−30	−48	−5	−39	−63	−43	0	−5
PT Metrics	6MWT Dist. (%)	+4.4	+16.3	+7.6	–	+1.9	−14.0	+12.9	−0.6	+8.8	+74.3	−34.7	−50.0	–	−39.7	–
	GSGC	0	0	0	–	1	−2	0	0	3	0	0	1	–	−2	–
	Gait (m/s)	+0.14	+0.04	+0.06	–	−0.09	+0.36	+0.28	+0.24	+0.31	+0.10	+0.47	−0.12	–	+0.04	–
	QMFT	+2	0	0	–	−1	+11	+5	+1	–	−5	−2	−3	–	+6	–
	GMFM (%)	–	–	0	–	–	+1.43	0	–	–	–	–	–	–	–	–
	BOT-2	–	–	–	–	–	–	+3	–	–	–	–	–	–	–	–
PFTs	FVC (%)	+6	−2	–	+16	–	–	–	+6	−8	−1	–	–	–	+2.4	–
PROs	Dyspnea	–	−5	−6	–	–	–	–	−4	–	–	−13	–	–	–	–
	Fatigue	–	−7	+3	–	–	–	–	−1	–	–	–	–	–	–	–
	PF	–	+1	+3	–	–	–	–	+3	–	–	−2	–	–	–	–
	LBP	–	−6	−3	–	–	–	–	−2	–	–	–	–	–	–	–

In particular, we highlight patient 13, who exhibited severe, progressive disease despite receiving 19 years of alglucosidase therapy up to a maximum dose of 40 mg/kg q1w, which she received for 2.5 years. She has now been on avalglucosidase alfa at 40 mg/kg q2w for a total of 18 months. Although she was not able to undergo the standard PT assessments, she demonstrated continued objective improvements in 9-HPT time and lateral pinch strength after switching to avalglucosidase. She also showed substantial improvement in her Hex4 biomarker. She noted considerable improvement of her motor function, breathing ability, endurance, and overall quality of life, as well as resolution of the infusion-associated reactions that she experienced while on alglucosidase. She has had physical and occupational therapy on and off throughout her life and was participating in both while on avalglucosidase; thus, it cannot be determined how much of her improvement was due to switching ERT and how much was due to PT/OT interventions. This is the first outcome data in a non-ambulatory Pompe patient with the switch from alglucosidase to avalglucosidase.

Some patients in this cohort with particularly severe clinical manifestations appeared to benefit from a higher dosage of avalglucosidase at 40 mg/kg q2w. P4, P8, and P13 transitioned to this dose directly from 40 mg/kg alglucosidase, at either q1w or q2w, and P7 increased to 40 mg/kg q2w after demonstrating a suboptimal response to 20 mg/kg q2w. In the management of patients with Pompe disease, there is often a need to utilize dosing beyond that recommended by the package insert to maximize benefit to the patient. These four patients showed improvement in clinical status without any adverse effects or safety concerns from the higher dosage. Further research into the safety and efficacy of increasing avalglucosidase dosage beyond standard recommendations is needed.

Laboratory markers (CK, Hex4, AST, ALT) improved in all patients at statistically significant levels from alglucosidase alfa to avalglucosidase alfa. Notably, while laboratory values did decrease substantially from baseline to alglucosidase alfa, a majority of those values remained above the upper limit of normal. Switching to avalglucosidase alfa was associated with further decreases in biomarkers, more than doubling the number that fell within the reference range for normal. These improvements in biomarkers were accompanied by evidence of improvement in clinical function measured in physical therapy metrics, PFTs, and PROs.

Improvement in motor and pulmonary function is a much sought-after outcome in the treatment of Pompe disease, and in this cohort, *n* = 4/7 patients had improvements in percent predicted upright FVC, while *n* = 3/7 showed a decline in percent predicted upright FVC. Unfortunately, without a true established MCIC for upright FVC in Pompe disease, interpretation of these changes is challenging. PFT data was limited, and patient 11 in particular only had two data values and no true historical baseline was able to be established for her. Notably, patient 5, the one patient with a decline in FVC, experienced an early improvement in percent predicted FVC from 91% to 97%, recorded around 18 months after switching. This improvement persisted at 28 months after the switch. However, the next set of PFTs at 54 months post-switch showed a decline, which progressed for the rest of her treatment history arriving at her most recent value of 83%. Her supine values followed a similar trend, decreasing from 67% pre-switch to 47% at most recent evaluation. Upon chart review, no direct cause for this decline could be identified. Despite worsening in PFTs, patient 5 did show improvement in laboratory markers and PT assessments.

Notably, those with mild-to-moderate functional impairments were the most likely to show improvement or stabilization in their PT scores, suggesting that the individuals with less established muscle damage retained greater capacity for functional improvement. A majority (*n* = 8/12) of patients have improved or remained stable in 6MWT distance since the switch. Of the four that did decline, three were among those with the most impaired gait, and one was a ten-year-old with low effort during the test (2/10 on the self-reported effort scale and minimal change in heart rate during the test, from 84 to 86 bpm).

Most (*n* = 9/12) patients stabilized or improved on the GSGC at their most recent visit, with additional positive trends noted in the timed functional sub-components of the GSGC. There were statistically significant improvements in comfortable and fast gait speed, while supine-to-stand (Gowers) and sit-to-stand (Chair) showed trends toward improvement that were not statistically significant. For gait speed, there were meaningful clinical improvements or stability for all but one patient at all time points after the switch. Gait speed is associated with various outcomes and functional categories (Middleton et al., 2015), thus stability or improvement in speed is important for functional capacity. A speed below 0.8 m/s is clinically significant, as it indicates that the patient may be categorized functionally as a limited community ambulator, while speeds below 0.42 m/s indicate that they are more likely a household only ambulator (Perry et al., 1995). Patients with severe functional impairments had the slowest walking speeds, yet one moved from the household-only ambulator category to limited-community ambulator (P6). The two patients in the moderately impaired group (P2, P5) had gait speeds between the mild and severe groups, and both made gains which placed them more firmly in the community ambulator category. All of the mildly impaired patients had gait speeds placing them in the community ambulator group, and they remained stable or improved.

QMFT scores also improved or stabilized in a majority (*n* = 7/11) of patients, with increases ranging from +1 to +11. No MCID has been established for the QMFT, limiting interpretation of the clinical significance of these data. GMFM and BOT-2 were available for fewer patients (*n* = 3 and *n* = 1, respectively), and they showed improvement or stabilization in all cases.

Finally, although PRO data was limited in this cohort, the majority of patients that had data at multiple timepoints showed improvements in dyspnea (*n* = 4/4), physical function (*n* = 3/4), fatigue (*n* = 2/3), and lower back pain (*n* = 3/3).

There were five reports of infusion reactions while receiving alglucosidase alfa and one report of infusion reactions after the switch to avalglucosidase alfa. While on alglucosidase alfa, *n* = 9/10 patients had positive ADA titers ranging from 400 to 12,800. While on avalglucosidase alfa, *n* = 8/13 patients had positive ADA titers ranging from 400 to 102,400. ADA peak titers ≥12,800 have been associated with an increased risk of infusion-associated reactions and a trend toward decreased pharmacodynamic response as measured by percent change in urinary Hex4 (Sanofi Genzyme, 8/2021). Two patients (patients 11 and 14) had sustained titers above 12,800.

Patient 11 has three ADA data points while on avalglucosidase alfa, and these are trending downward (102,400, 51,200, 25,600). Assessments in this patient since switching include PFTs and biomarkers. On PFTs, she was limited to two data points without a clear baseline, increasing from 82% to 98%. Biomarkers showed improvements of 11% in CK and AST, both of which normalized, as well as an improvement in Hex4, which has remained within normal limits. Other than two episodes of fever associated with infusions, she has had no infusion-associated reactions.

Patient 14 had two ADA data points (12,800 and 25,600) since switching to avalglucosidase alfa. Initial follow-up data in this patient includes PT assessments and biomarkers. He showed stability in 6MWT and gait speed, with a worsening of GSGC and QMFT by one point each. Biomarkers in this patient showed improvements of 31% in CK, 56% in Hex4, and 24% in AST. CK (657 U/L) and AST (42 U/L) remain above the upper limit of normal, while Hex4 normalized. He has had no infusion-associated reactions.

Overall, initial post-switch assessments in patients 11 and 14 present a mixed bag of outcomes, and there is no clear clinical impact of these elevated titers as of yet. Continued close follow-up is necessary to evaluate their effects.

Limitations of this study include its relatively small cohort (*n* = 15) and short follow-up period. Many of the patients did not complete all of the evaluations used therein, and consequently some draw upon limited amounts of data. For instance, longitudinal PRO outcomes were only available in *n* = 4 patients; this was largely due to a lack of consistent data obtainable during the COVID-19 pandemic. Moreover, the selection of patients took place via convenience sampling, utilizing patients at the Duke metabolic clinic who met the inclusion criteria of switching ERT regimens. While this sample included a variety of patients, it may not be statistically representative of the general population of patients with LOPD. Despite thorough and comprehensive review of patient charts for the abstraction of data, it is possible that some data was missing from the chart or otherwise unavailable for use in this study. Future studies should be oriented toward substantiating these findings across a larger cohort of patients and with stricter control of confounding variables. The short follow-up periods resulted in data from only a few follow-up visits after the switch. This was especially apparent with patient 16, who had PT follow up only at 72-days post-switch. Continued follow-up is necessary to fully understand how patients are faring. This study also applied the MCID from other chronic pulmonary diseases to FVC results in LOPD, a common but imperfect practice that may not adequately reflect the pathological process in Pompe disease (Lachmann and Schoser, 2013). Finally, it should be noted that many of the PT evaluations were performed by different PTs. Although all examiners were experienced with Pompe disease, some discrepancies between scores, especially QMFT, were seen between different evaluators, reflecting the potentially decreased inter-rater reliability in a real-world clinical setting compared to a research setting.

In conclusion, many patients in this cohort stabilized or improved after the switch from alglucosidase alfa to avalglucosidase alfa. A subset of patients with particularly severe manifestations appeared to show more persistent decline. Stabilization already represents a much-improved outcome compared to the well-documented natural history of progressive decline in LOPD, but many patients in this cohort also showed tangible improvements after the switch (Hagemans et al., 2005; Winkel et al., 2005; Dimachkie et al., 2022). Overall, stabilization or improvement was seen across several different outcome metrics, including laboratory markers (*n* = 15/15), PT evaluations (*n* = 8/12 in 6MWT, *n* = 9/12 in GSGC, *n* = 11/12 in gait speed, and 7/12 in QMFT), PFTs (*n* = 4/7), and PROs (*n* = 3/4 in physical function or *n* = 4/4 in dyspnea, fatigue, and back pain). This study presents additional evidence that switching from alglucosidase to avalglucosidase may be associated with improved outcomes in a subset of patients with LOPD.

## Data Availability

The original contributions presented in the study are included in the article/Supplementary Material, further inquiries can be directed to the corresponding author.
