# Supplementary material for: Real-world outcomes from a series of patients with late onset Pompe disease who switched from alglucosidase alfa to avalglucosidase alfa
Source: Front Genet. 2024 Jan 19;15:1309146. doi: 10.3389/fgene.2024.1309146 (PMC10834735; doi:10.3389/fgene.2024.1309146)

Supplementary Material

1.1 Supplementary Tables

1.1.1 **Supplementary Table 1.** CK and AST trends. Baseline values are the last known values prior to initiating ERT. Alglucosidase values are the last known values prior to the switch. Avalglucosidase values are the most recent values available in the chart. Other timepoints were taken into account when selecting these to ensure that general trends were reflected. Full data is available in Supplementary Figure 1.

	**CK** (30-220 U/L)			**AST** (15-41 U/L)		
**Patient/Sex**	Baseline	Alg.	Aval.	Baseline	Alg.	Aval.
1/F	541	337	158	56	43	29
2/F	–	278	143	–	30	21
3/F	539	429	382	45	42	34
4/M	301	138	123	38	21	20
5/F	614	374	152	–	60	31
6/F	–	195	83	–	28	17
7/F	–	211	200	–	88	33
8/M	235	209	205	45	47	27
9/M	470	271	188	52	30	26
11/F	468	242	216	59	42	26
12/F	269	210	83	28	19	19
13/F	–	–	14	–	21	20
14/M	1992	955	657	87	55	42
15/M	950	336	232	305	67	42
16/M	966	913	–	315	61	–

1.1.2 **Supplementary Table 2.** ALT and Hex4 Trends. Baseline values are the last known values prior to initiating ERT. Alglucosidase values are the last known values prior to the switch. Avalglucosidase values are the most recent values available in the chart. Other timepoints were taken into account when selecting these to ensure that general trends were reflected. Full data is available in Supplementary Figure 1.

	**ALT** (5-40 U/L)			**Hex4** ($\leq$3.0 mmol/mol Cr)		
**Patient/Sex**	Baseline	Alg.	Aval.	Baseline	Alg.	Aval.
1/F	72	53	32	–	4.2	1.83
2/F	–	24	28	–	4.7	2.8
3/F	45	38	30	8.09	2.6	1.1
4/M	32	22	18	2.9	2.2	1.8
5/F	–	67	34	8	4.6	1.6
6/F	–	17	11	–	3.5	2
7/F	–	129	30	–	4.8	3.6
8/M	91	44	26	8.7	4.5	–
9/M	60	36	23	4.3	1.6	1.4
11/F	35	24	14	3.2	2.4	0.9
12/F	53	44	30	10.7	8.5	4.4
13/F	–	42	23	–	25	5.7
14/M	74	53	42	4	3.9	1.7
15/M	233	95	38	–	5	2.6
16/M	285	52	–	–	3.2	2.8

1.1.3 **Supplementary Table 3.** Anti-alglucosidase Antibody Titers. Most recent and peak anti-alglucosidase antibody titers with months before switch to avalglucosidase.

Patient/Sex	Most Recent Alglucosidase	Months Before Switch	Peak Alglucosidase	Months Before Switch
1/F	Negative	2.69	3200	16.95
2/F	Negative	3.68	3200	30.82
3/F	Negative	2.30	Negative	2.30
4/M	800	0.03	6400	21.59
5/F	ND	ND	ND	ND
6/F	12800	2.50	12800	2.50
7/F	400	3.94	400	3.94
8/M	Negative	1.51	1600	64.49
9/M	200	4.11	200	4.11
11/F	ND	ND	ND	ND
12/F	800	2.63	800	2.63
13/F	ND	ND	ND	ND
14/M	6400	3.71	6400	3.71
15/M	ND	ND	ND	ND
16/M	ND	ND	ND	ND

1.1.4 **Supplementary Table 4.** Anti-avalglucosidase Antibody Titers. Most recent and peak anti-avalglucosidase antibody titers with months since switch to avalglucosidase.

Patient/Sex	Most Recent Avalglucosidase	Months After Switch	Peak Avalglucosidase	Months After Switch
1/F	Negative	9.27	Negative	9.27
2/F	Negative	8.28	Negative	8.28
3/F	800	3.32	800	3.32
4/M	800	11.99	800	11.99
5/F	3200	108.22	3200	108.22
6/F	Negative	9.46	Negative	9.46
7/F	Negative	51.25	3200	34.04
8/M	ND	ND	ND	ND
9/M	400	3.65	400	3.65
11/F	25600	20.34	102400	11.04
12/F	Negative	33.25	400	3.02
13/F	Negative	9.53	Negative	9.53
14/M	25600	12.39	25600	12.39
15/M	Negative	10.91	Negative	10.91
16/M	ND	ND	ND	ND

1.1.5 **Supplementary Table 5.** Quick Motor Function Test (QMFT). Changes in QMFT total score (green improved, blue stable, red declined) with additional breakdown by task item. No MCD or MCID has been established for this test. Scores range from 0 to 64, with a score of 64 indicating normal function.

**Patient ID**	**1F**	**3F**	**9M**	**14M**		**15M**	**16M**	**2F**		**4M**			**6F**		**7F**			**12F**	
**Severity (per GSGC)**	Mild	Mild	Mild	Mild		Mild	Mild	Mod		Sev			Sev		Sev			Sev	
**Days after switch**	282	101	475	100	377	333	72	81	252	162	365	572	103	288	366	538	1133	971	1376
**Total Score**	**50**	**63**	**50**	**60**	**60**	**64**	**64**	**32**	**33**	**18**	**17**	**16**	**29**	**27**	**14**	**13**	**11**	**24**	**27**
**Change from baseline**	**2**	**0**	**0**	**-1**	**-1**	**11**	**5**	**0**	**1**	**-3**	**-4**	**-5**	**0**	**-2**	**0**	**-1**	**-3**	**3**	**6**
**Raising torso**						1					-1			1					
**Neck flex**						1				-1							-1	1	
**Hand across midline**																	-1	1	
**Hip and knee flex**									-1					1		-1		1	
**Extend the legs**						1	1												
**Sit up**				-1		2												-1	
**Extend the arms**						1								-1				-2	2
**Stand from chair**																			
**Stand from half knee**						1	1												
**Squat**						1	1												
**Stand from squat**						1	1												
**Pick up object**	1								1					-1				2	
**Single leg stand**						1	1		1					-1				1	1
**Walking**												-1		-1					
**Jumping**	1					1													
**Stairs**										-2									

1.1.6 Supplementary Table 6. 9-hole peg test (9-HPT) results for Patient 13F. Times in seconds for completion of the standard or modified easy grip 9-HPT are listed for patient 13F prior to and after the switch to avalglucosidase alfa.

Days from switch	-184	-44	12	286	433	518
**Easy grip 9-HPT (right hand)**	118	110	87	57	–	–
**Standard 9-HPT (right hand)**	Unable	Unable	Unable	98	78	58
**Easy grip 9-HPT (left hand)**	245	162	110	59	–	–
**Standard 9-HPT (left hand)**	Unable	Unable	Unable	Unable	88 for 6/9 pegs	116

1.1.7 Supplementary Table 7. Comfortable gait speeds at each visit before and after the switch from alglucosidase to avalglucosidase. Items in blue met the MDC of 0.15 m/s and the lower MCID cutoff of 18.9% and those in blue with green outline met the most stringent MCID of 31.1%. Two patients (4M and 7F) had changes that were significant using both MCID cutoffs at their most recent visits, but their baseline walking speeds were so slow that these cutoffs were lower than the MDC of 0.15 m/s; patient 4M (in green) met both MCID cutoffs for significant improvement, and patient 7F (in red) met both MCID cutoffs for significant decline.

		Comfortable Gait Speed (m/s)							Change in Gait Speed from baseline to:				
Patient	Severity based on GSGC	2 Prior	1 Prior	1 After	2 After	Most recent	At Best	At Worst	1 After	2 After	Most recent	Best after	Worst after
15/M	Mild		0.93	1.29		1.29	1.29	1.29	0.36		0.36	0.36	0.36
3/F	Mild		1.36	1.40		1.40	1.40	1.40	0.04		0.04	0.04	0.04
9/M	Mild	1.30	1.32	1.18	1.38	1.38	1.38	1.18	-0.14	0.06	0.06	0.06	-0.14
16/M	Mild	1.04	1.11	1.39		1.39	1.39	1.39	0.28		0.28	0.28	0.28
14/M	Mild	1.23	1.50	1.45	1.41	1.41	1.45	1.41	-0.05	-0.09	-0.09	-0.05	-0.09
1/F	Mild	1.29	1.19	1.33		1.33	1.33	1.33	0.14		0.14	0.14	0.14
5/F	Mod	1.52	0.88	0.95	1.30	1.19	1.30	0.76	0.07	0.42	0.31	0.42	-0.12
2/F	Mod	0.93	0.96	0.97	1.20	1.20	1.20	0.97	0.01	0.24	0.24	0.24	0.01
6/F	Severe	0.22	0.24	0.64	0.61	0.71	0.71	0.61	0.4	0.37	0.47	0.47	0.37
7/F	Severe		0.32	0.34	0.33	0.20	0.34	0.20	0.02	0.01	-0.12	0.02	-0.12
4/M	Severe	0.38	0.32	0.35	0.41	0.42	0.41	0.35	0.03	0.09	0.10	0.10	0.03
12/F	Severe		0.61	0.80	0.65	0.65	0.80	0.65	0.19	0.04	0.04	0.19	0.04
**Average speed**			**0.90**	**1.01**	**0.84**	**1.05**	**1.08**	**0.96**					

1.2 Supplementary Figures

1.2.1 **Supplementary Figure 1. Full lab trends.** All lab data available for CK, AST, and Hex4 graphed for all 15 patients by age. Color-coded vertical bars represent the starting points of ERT regimens, as described in Figures 1 and 3.

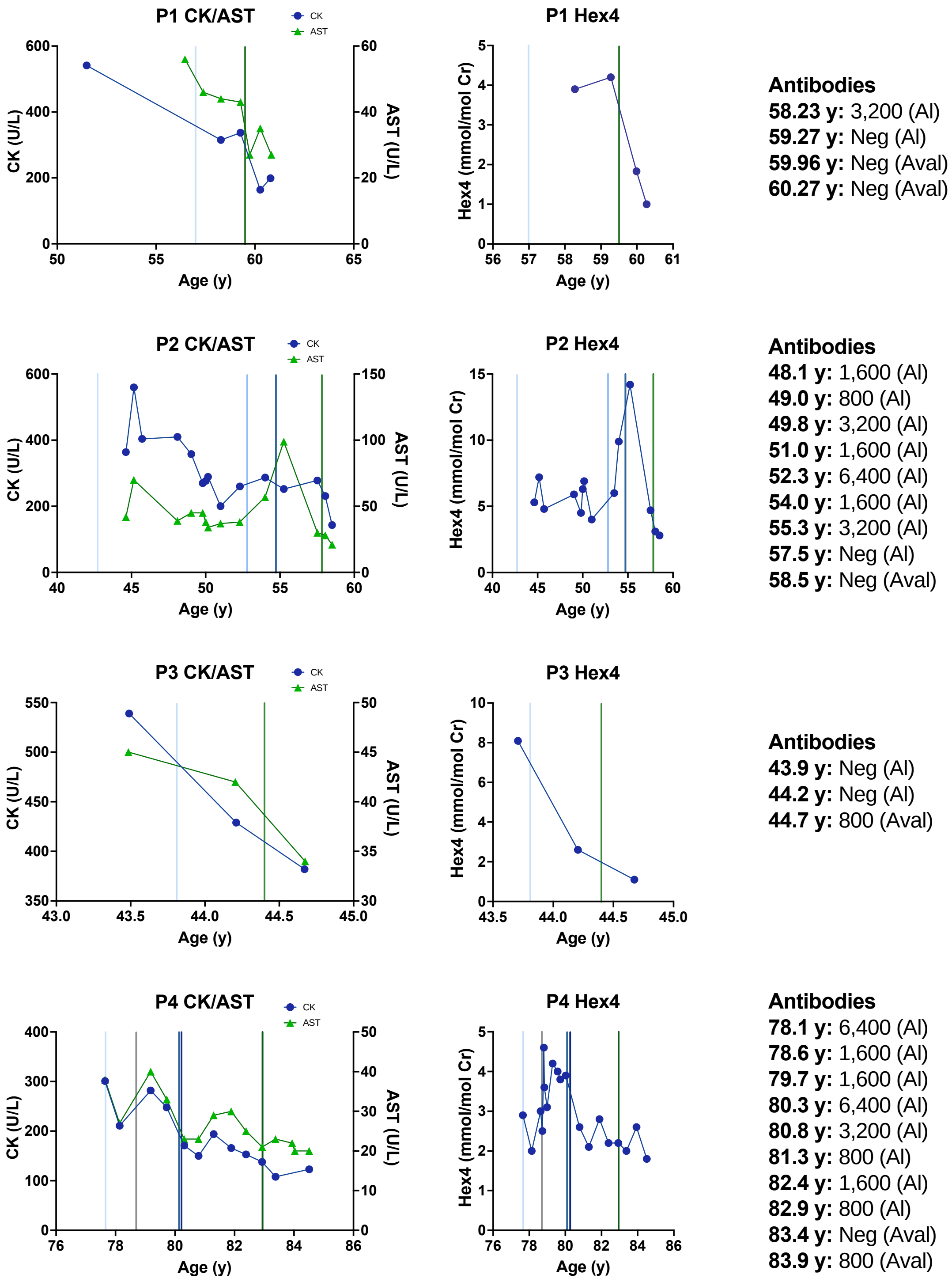

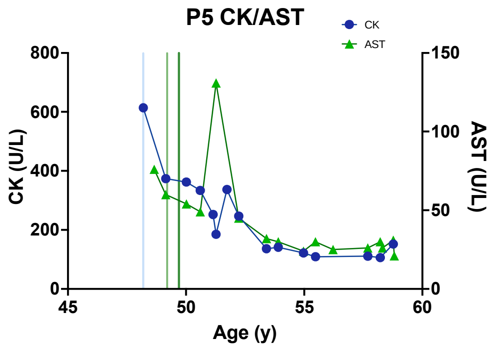

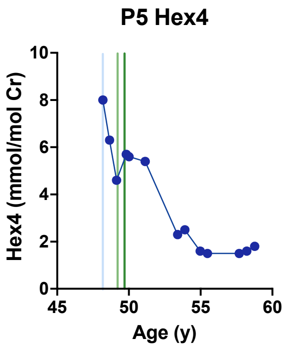

P5 Antibodies

48.7 y: 800 (Al)
49.1 y: 1,600 (Al)
52.2 y: 1,600 (Al)
53.4 y: 1,600 (Al)
53.9 y: 1,600 (Al)
57.7 y: 1,600 (Aval)
58.2 y: 3,200 (Aval)

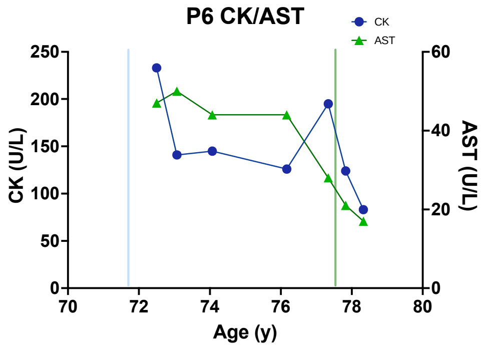

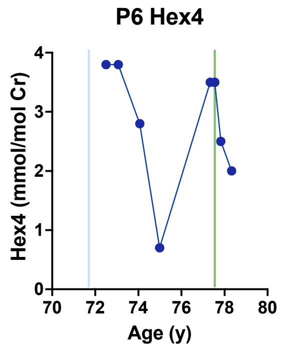

P6 Antibodies

72.5 y: 800 (Al)
73.0 y: 12,800 (Al)
74.1 y: 6,400 (Al)
75.0 y: 12,800 (Al)
77.3 y: 12,800 (Al)
77.8 y: Neg (Aval)
78.3 y: Neg (Aval)

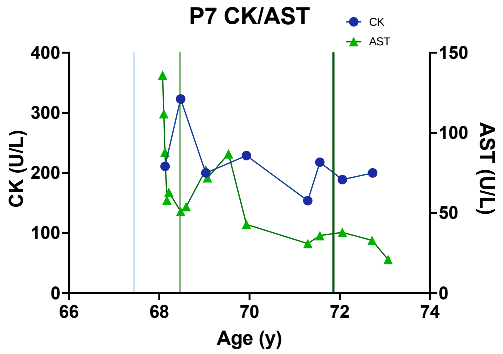

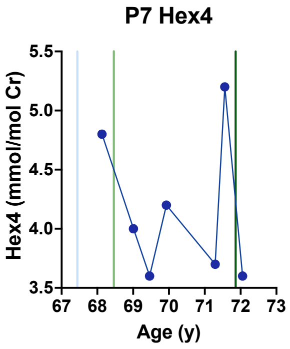

P7 Antibodies

68.1 y: 400 (Al)
68.5 y: 400 (Al)
71.3 y: 3,200 (Al)
72.1 y: 1,600 (Aval)
72.7 y: Neg (Aval)

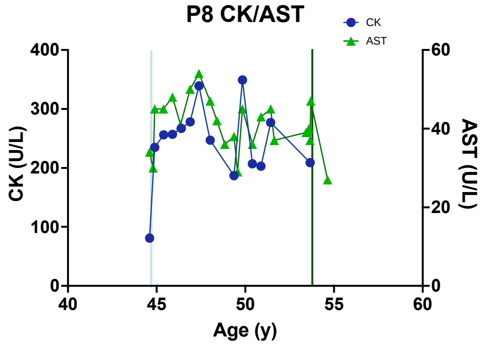

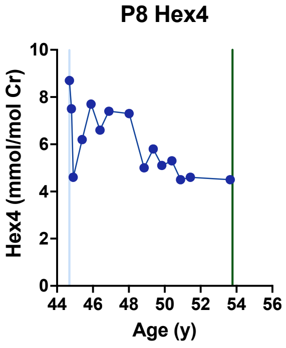

P8 Antibodies

44.9 y: 200 (Al)
45.4 y: 200 (Al)
46.9 y: 400 (Al)
47.4 y: 1,600 (Al)
48.0 y: 1,600 (Al)
49.4 y: 400 (Al)
49.8 y: 100 (Al)
50.4 y: 800 (Al)
50.9 y: 1,600 (Al)
51.4 y: Neg (Al)
53.7 y: Neg (Al)

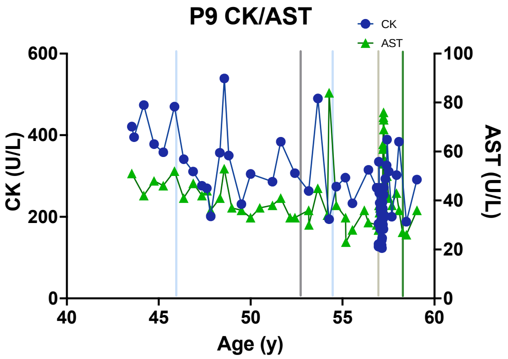

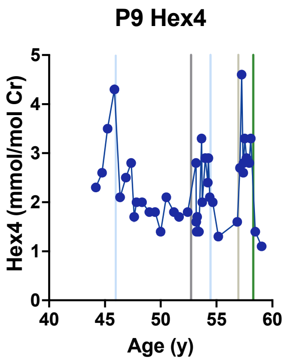

P9 Antibodies

47.6 y: 6,400 (Al)
47.7 y: 12.800 (Al)
47.8 y: 6,400 (Al)
48.3 y: 12,800 (Al)
48.6 y: 6,400 (Al)
48.8 y: 6,400 (Al)
49.0 y: 6,400 (Al)
49.5 y: 1,600 (Al)
50.5 y: 1,600 (Al)
51.6 y: 1,600 (Al)
53.2 y: 1,600 (Al)
54.7 y: 3,200 (Al)
55.2 y: 800 (Al)
55.5 y: 400 (Al)
56.4 y: 200 (Al)
56.9 y: 400 (Al)
57.0 y: 200 (Al)
57.1 y: 200 (Al)
57.2 y: 200 (Al)
57.4 y: 200 (Al)
57.5 y: 200 (Al)
57.7 y: 200 (Al)
57.9 y: 200 (Al)
58.5 y: 400 (Aval)
58.6 y: 400 (Aval)
59.0 y: 400 (Aval)

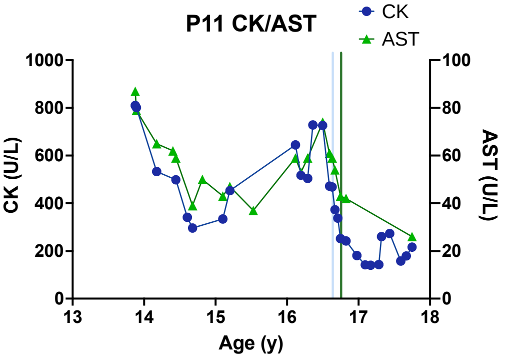

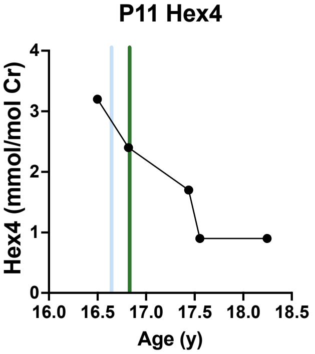

P11 Antibodies

17.8 y: 102,400 (Aval)
18.1 y: 51,200 (Aval)
18.5 y: 25,600 (Aval)

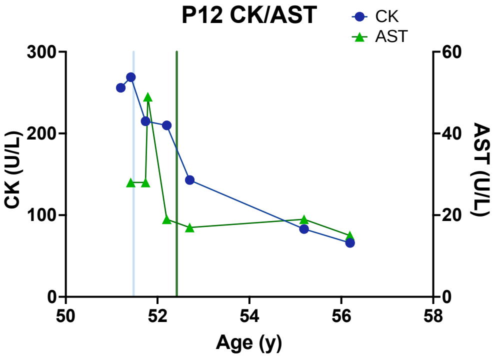

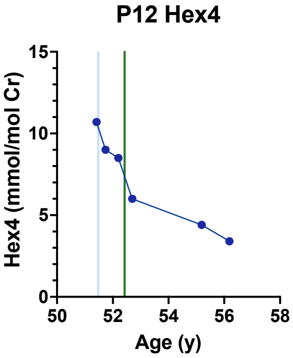

P12 Antibodies

51.7 y: 200 (Al)
52.2 y: 800 (Al)
52.7 y: 400 (Al)
55.2 y: Neg (Aval)
56.2 y: Neg (Aval)

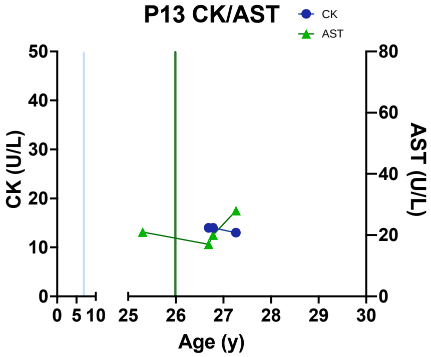

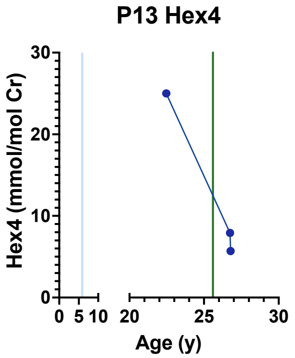

Antibodies

26.8 y: Neg (Aval)

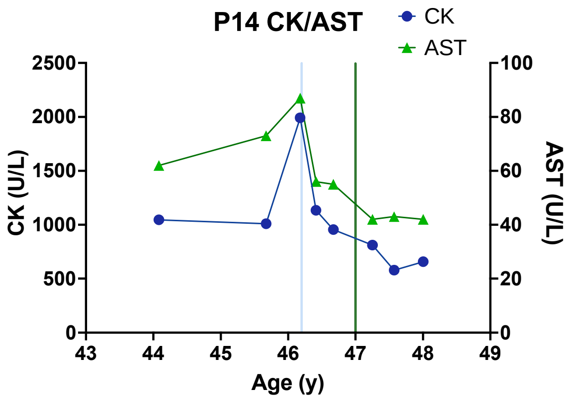

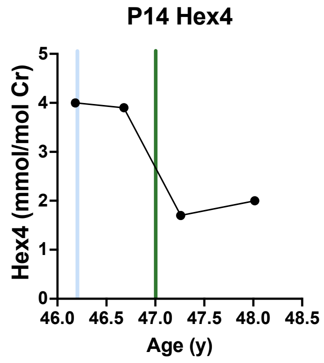

Antibodies
46.7 y: 6,400 (Al)
47.3 y: 12,800 (Aval)
48.0 y: 25,600 (Aval)

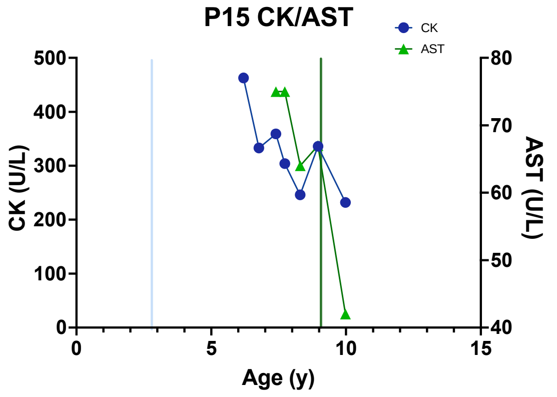

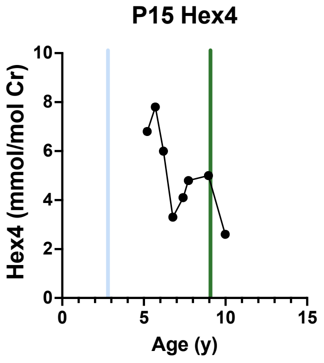

Antibodies
10.0 y: Neg (Aval)

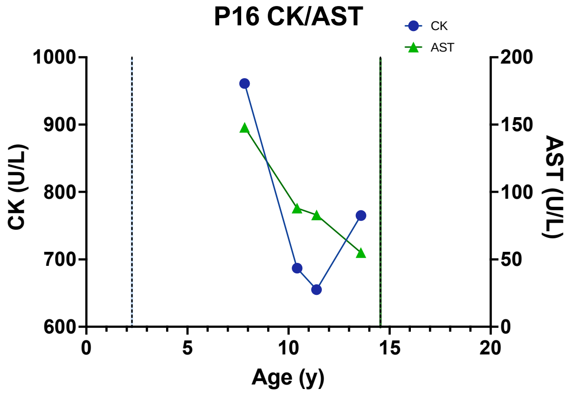

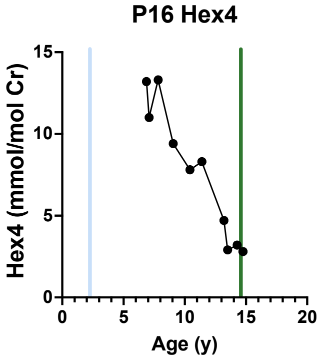

Antibodies
7.8 y: 200 (Al)
10.4 y: <100 (Al)
11.4 y: 200 (Al)

1.2.2 **Supplementary Figure 2.** Six-Minute Walk Test (6MWT). Above: an overview of 6MWT percent-predicted at evaluations before and after the switch. Colors are based on baseline GSGC level of functional impairment [mildly impaired in green (n=6), moderately impaired in yellow (n=2), and severely impaired in red (n=4)]. Below: full data of individual scores over time. Color-coded vertical bars represent the starting points of ERT regimens, as described in Figures 1 and 3.

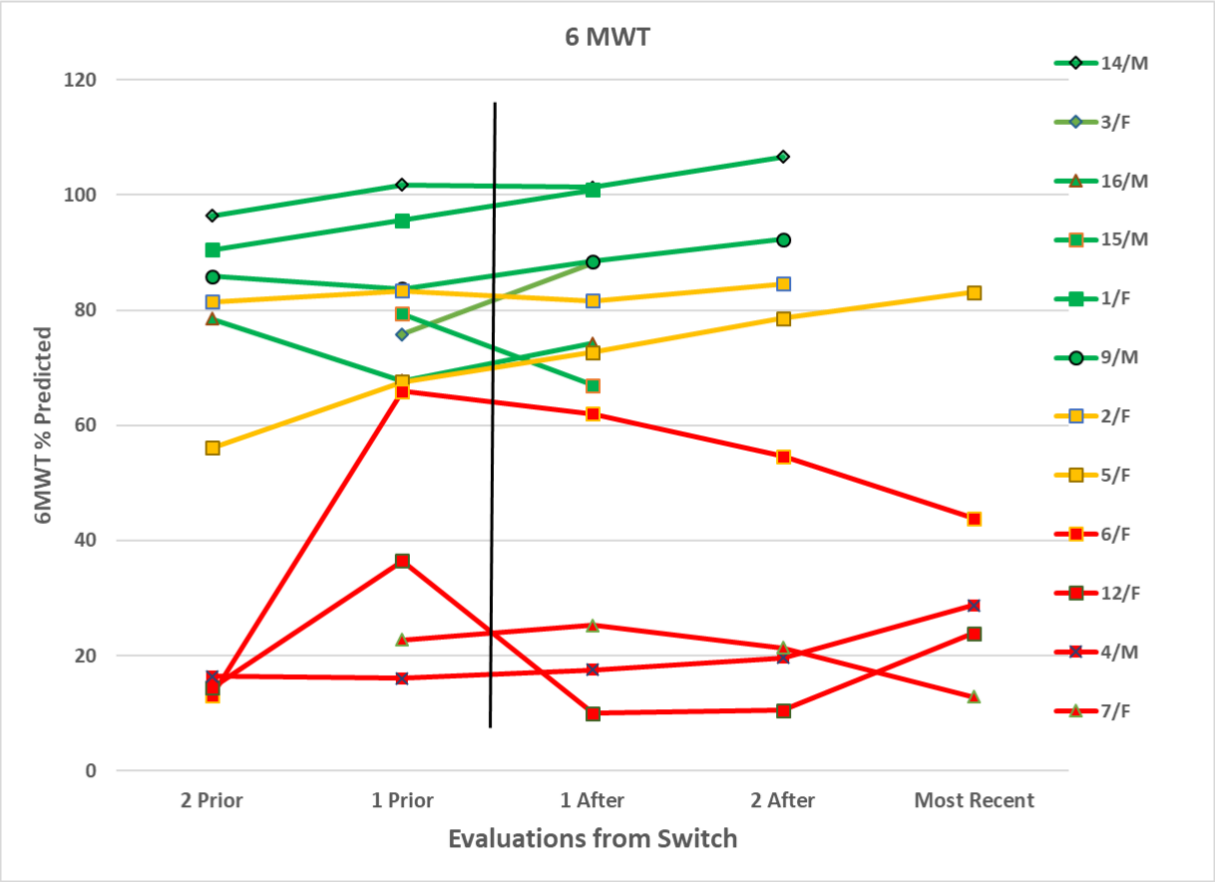

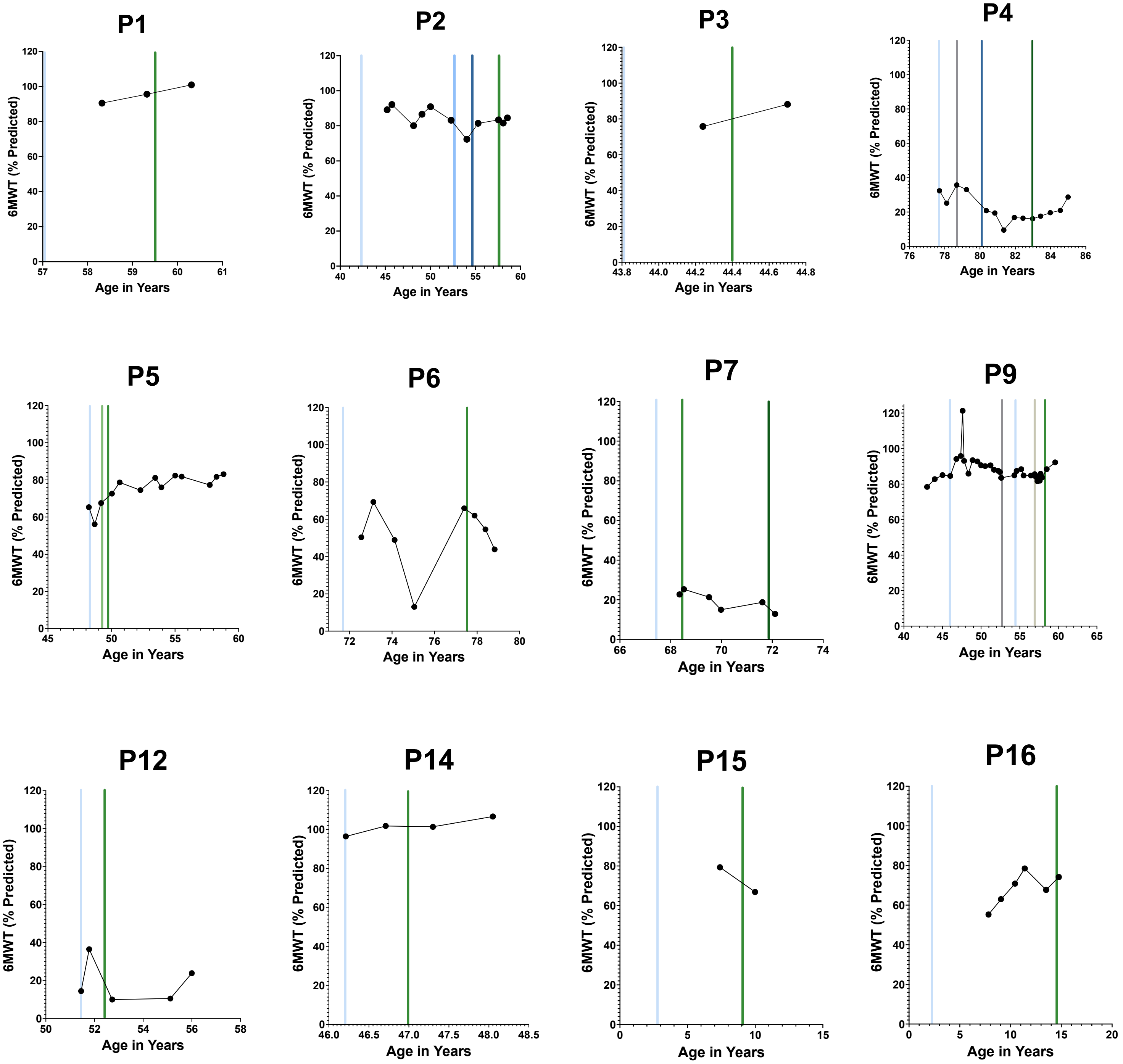
P1
P2
P3
P4
P5
P6
P7
P9
P12
P14
P15
P16
6MWT (% Predicted)
Age in Years

1.2.3 **Supplementary Figure 3.** Gait, Stairs, Gower, Chair (GSGC). Above: an overview of GSGC scores at timepoints before and after the switch. For patients seen more than twice after the switch, the most recent score was used for the last time point. GSGC scores range from 4 to 27 with score of 4 indicating normal function. Colors are based on baseline GSGC level of functional impairment [mildly impaired in green (n=6), moderately impaired in yellow (n=2), and severely impaired in red (n=4)]. Below: full data of individual scores over time. Color-coded vertical bars represent the starting points of ERT regimens, as described in Figures 1 and 3.

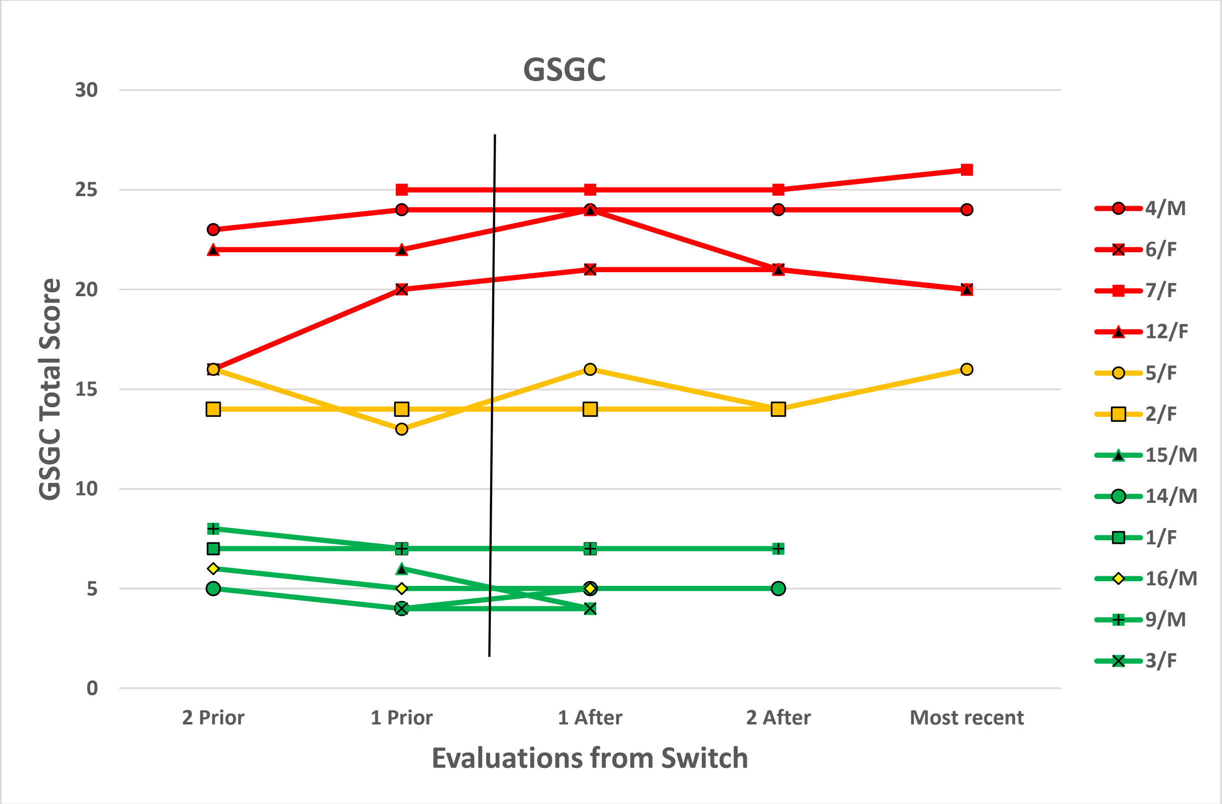

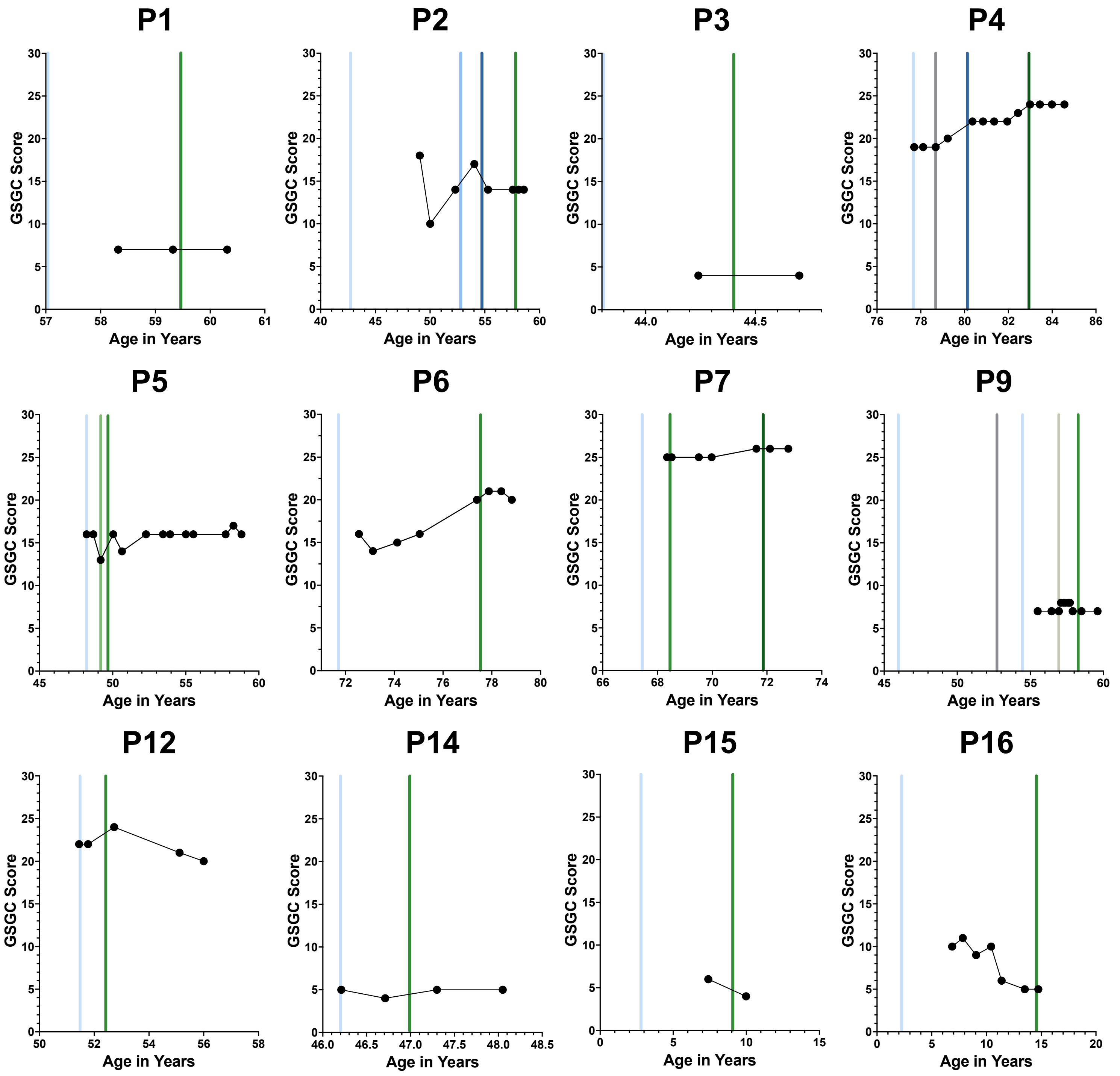
P1
P2
P3
P4
P5
P6
P7
P9
P12
P14
P15
P16
GSGC Score
Age in Years

1.2.4 **Supplementary Figure 4.** Comfortable Gait Speed. Gait speed expressed in meters per second at timepoints before and after the switch. Colors are based on baseline GSGC level of functional impairment [mildly impaired in green (n=6), moderately impaired in yellow (n=2), and severely impaired in red (n=4)]. The minimal detectable change used for gait speed was 0.15 m/s.

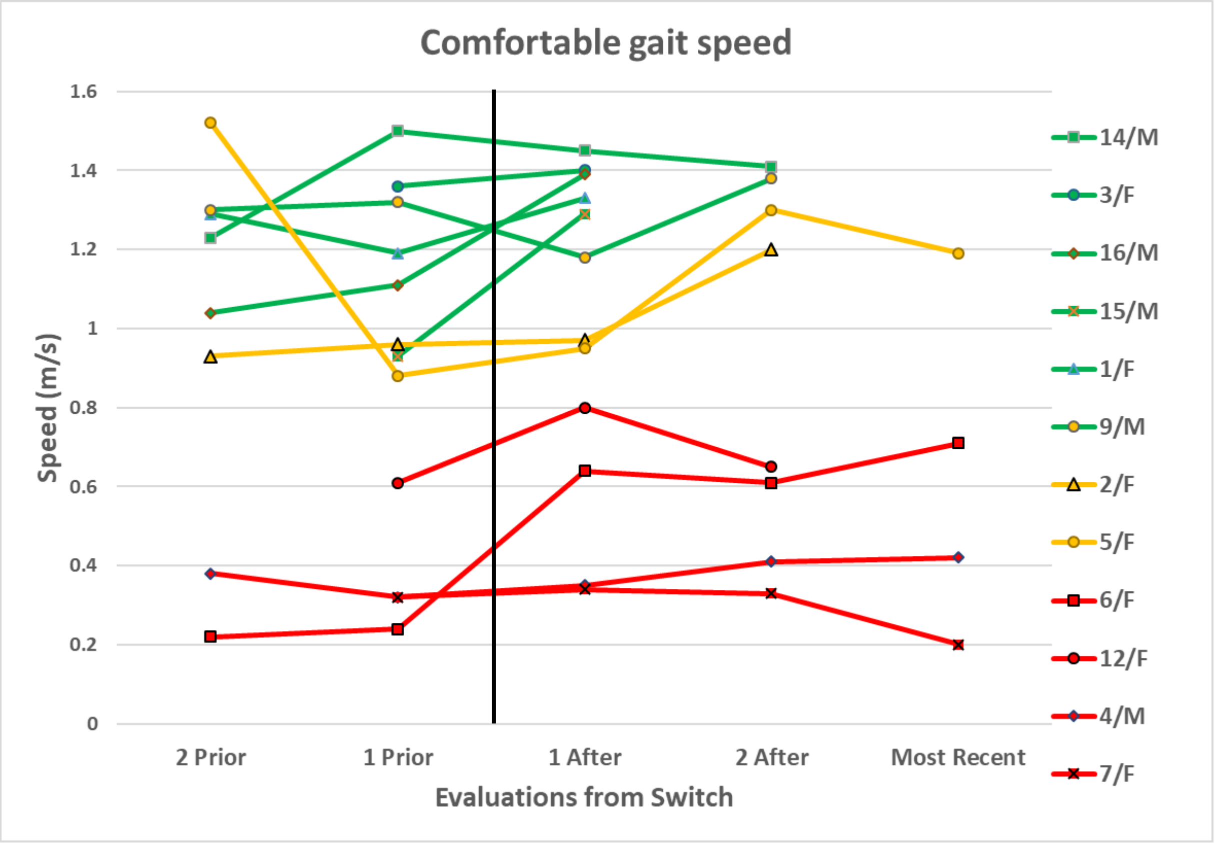

1.2.5 **Supplementary Figure 5 (A-E).** Gait Speed, Gowers (supine to stand), and Chair (sit to stand) comparisons and paired t-tests for timed values from baseline (pre) to most recent assessment or best post-switch assessment after switching to avalglucosidase alfa.

Overall, there were trends toward improvement in mean comfortable gait speed, fast gait speed, supine to stand (Gowers), and sit to stand (Chair) at the most recent and best post-switch assessments. The mean increase in comfortable gait speed and fast gait speed was statistically significant at both most recent and best post-switch assessments at an alpha level of 0.05. The mean Gowers (supine to standing) and sit to stand both showed statistically significant improvements at an alpha level of 0.05 only when comparing pre-switch values to best post-switch values.

In the 6MWT (not depicted here), there was a trend towards improvement when comparing pre-switch values to the best post-switch performances, but it was not statistically significant.

A. Self-Selected Gait Speed – Pre vs most recent. The mean self-selected (comfortable) gait speed was faster post-switch for both most recent and best post-switch visits (mean 0.90 m/s pre-switch, 1.03 m/s at most recent and 1.07 m/s at best post-switch visit), showing a statistical significance at the alpha level of 0.05 at most recent and best post-switch assessments, analyzed with paired t-tests.

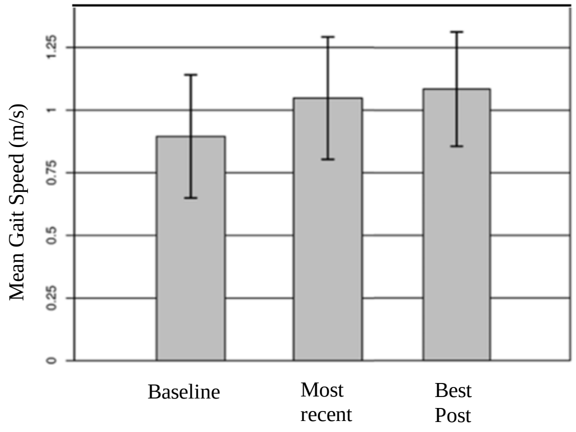

Self-selected (Comfortable) gait speed - pre		Self-selected (Comfortable) gait speed - most recent				
M	*SD*	*M*	*SD*	*t*	*p*	*d*
0.90	0.43	1.05	0.43	-2.91	.014	0.84

Note. N = 12. Degrees of Freedom for the *t*-statistic = 11. *d* represents Cohen's *d*.

Self-selected (Comfortable) gait speed - pre		Self-selected (Comfortable) gait speed - best				
M	*SD*	*M*	*SD*	*t*	*p*	*d*
0.90	0.43	1.08	0.40	-3.92	.002	1.13

Note. N = 12. Degrees of Freedom for the *t*-statistic = 11. *d* represents Cohen's *d*.

B. Fast gait speed - The mean fast gait speed was faster post-switch for both most recent and best post-switch visits (mean 2.13 m/s pre-switch, 2.48 m/s at most recent and at best post-switch visit), showing a statistical significance at the alpha level of 0.05, analyzed with paired t-tests.

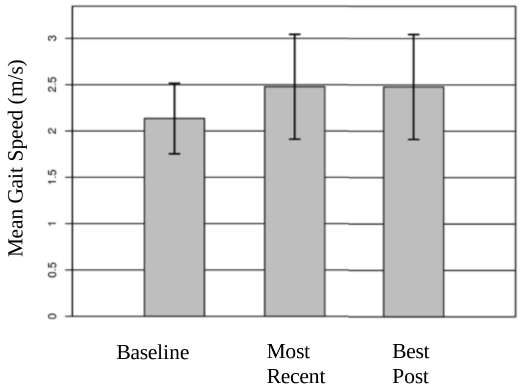

Fast gait speed - pre		Fast gait speed – most recent				
M	*SD*	*M*	*SD*	*t*	*p*	*d*
2.13	0.51	2.48	0.76	-2.58	.042	0.98

Note. N = 7. Degrees of Freedom for the *t*-statistic = 6. *d* represents Cohen's *d*.

Fast gait - pre		Fast gait - best				
M	*SD*	*M*	*SD*	*t*	*p*	*d*
2.13	0.51	2.48	0.76	-2.58	.042	0.98

Note. N = 7. Degrees of Freedom for the *t*-statistic = 6. *d* represents Cohen's *d*.

C. Gowers (supine to stand) – Pre vs best post-switch (t-tests)
Mean time to rise from supine to standing (Gowers) was faster post-switch (mean 7.85s pre-switch vs 7.02s at most recent and 6.61s at best post-switch), but was only statistically significant at an alpha level of .05 for the best post-switch visit, analyzed with paired t-tests.

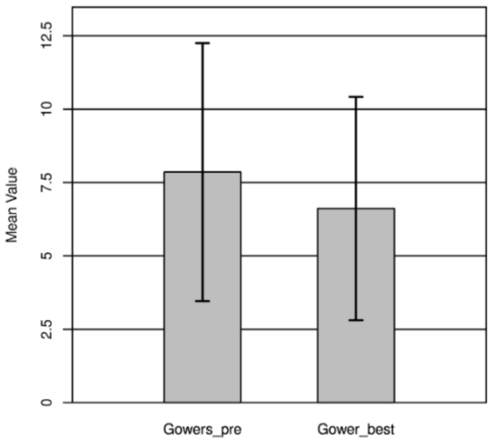

Gowers - pre		Gower - best				
M	*SD*	*M*	*SD*	*t*	*p*	*d*
7.85	6.34	6.61	5.49	2.42	.046	0.85

Note. N = 8. Degrees of Freedom for the *t*-statistic = 7. *d* represents Cohen's *d*.

D. Chair (sit to stand) – Pre vs best post-switch (t-tests)
Mean time to perform sit to stand task was faster post-switch (mean 2.39 s pre-switch vs 2.02 s at most recent and 1.58 s at best post-switch) but was only statistically significant at an alpha level of .05 for the best post-switch visit, analyzed with paired t-tests and a two-tailed Wilcox signed rank test.

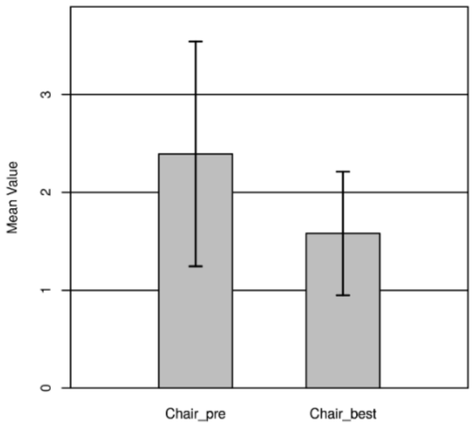

Chair - pre		Chair - best				
M	*SD*	*M*	*SD*	*t*	*p*	*d*
2.39	1.85	1.58	1.02	2.88	.018	0.91

Note. N = 10. Degrees of Freedom for the *t*-statistic = 9. *d* represents Cohen's *d*.

E. Chair (sit to stand) – Pre vs best post-switch (two-tailed Wilcoxon signed rank test)

A two-tailed Wilcoxon signed rank test, a non-parametric alternative to the paired samples t-test which does not share its distributional assumptions, was used because the normality assumption was violated, and was also significant based at best post-switch at an alpha value of .05, V = 54.00, z = -2.70, p = .007 which indicates that the differences are not likely due to random variation.

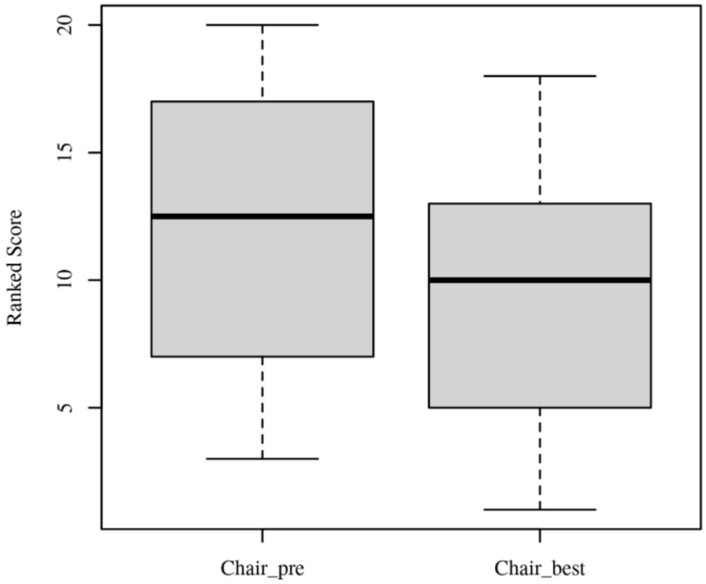

Supplement: Supplementary file 1 [file DataSheet1.pdf]
